# The Sensory and Physicochemical Properties of Honeybush Tea Depend on the Brewing Water: A Preliminary Study

**DOI:** 10.1111/1750-3841.70720

**Published:** 2025-11-29

**Authors:** Helene van Schoor, Erika Moelich, Brigitte von Pressentin du Preez, Chantelle Human, Dalene de Beer, Elizabeth Joubert

**Affiliations:** ^1^ Plant Bioactives Group, Post‐Harvest and Agro‐Processing Technologies Agricultural Research Council (Infruitec‐Nietvoorbij) Stellenbosch South Africa; ^2^ Department of Food Science Stellenbosch University Stellenbosch South Africa

## Abstract

This study investigated how different water types affected honeybush (*Cyclopia* species) tea quality. Water types, varying in physicochemical properties, were used to brew tea from three honeybush species: *C. intermedia*, *C. subternata*, and *C. genistoides* (n = 6/species). The water types were deionized water (W1), two bottled spring waters (W2, W3), bottled reverse osmosis water (W4), brackish water (W5), and tap water (W6). Descriptive sensory analysis was used to determine whether water type affected the sensory profile of the infusions. Other parameters included CIEL*a*b* color and turbidity, as well as the soluble solids and phenolic content of the infusions. Results showed that water with the highest pH (W3; pH = 8.3) and high EC (W3 and W5; EC >10 mS/m) negatively impacted the sensory profile of honeybush tea. Infusions made with W3 and W5 had a ‘date pudding’ aroma, which was uncharacteristic of honeybush, and lower intensities of typical floral and sweet‐associated honeybush aromas. These water samples also led to a salty taste, thick mouthfeel, and much darker color. Additionally, the concentrations of phenolic compounds, such as 3‐β‐D‐glucopyranosyl‐4‐*O*‐β‐D‐glucopyranosyliriflophenone, mangiferin, and isomangiferin, were reduced. Infusions prepared with W2, W4, and W6 (pH between 5.3 and 7.8; EC <10 mS/m) showed similar sensory and physicochemical profiles to those prepared with deionized water (pH = 7.1; EC = 0 mS/m).

**Practical Applications:**

For the best‐tasting honeybush tea, consumers should use water treated by reverse osmosis, with guaranteed low mineral content. This information will help producers and tea merchants present the true flavor of honeybush tea during tastings, supporting effective marketing.

## 1 Introduction

Honeybush herbal tea has gained global popularity due to its caffeine‐free nature and the presence of many polyphenolic compounds with health‐promoting properties (Joubert et al. 2019). Several *Cyclopia* species are used to produce honeybush tea, with the main product variant being “fermented” (oxidized) honeybush, produced using a high‐temperature oxidation process (Joubert et al. 2019). The characteristic sensory profile of the hot water infusion includes “fynbos‐floral,” “fynbos‐sweet,” “fruity‐sweet,” and “woody” aroma notes, a sweet taste, and a slightly astringent mouthfeel (Du Preez et al. 2020). Its sensory profile is a key parameter in establishing the quality of the infusions, together with its clarity and color (Du Preez 2020). Maintaining the characteristic sensory profile of honeybush tea is vital to ensure consumer confidence in the product.

In tea and herbal tea research, much emphasis has been placed on determining the effects of factors such as water temperature, steeping time, and water‐to‐leaf ratio on the overall quality of infusions (Cao et al. 2021; Franks et al. 2019; Ma et al. 2024; Muller et al. 2020; Murugesh et al. 2017; Xu et al. 2017). However, the quality of the water used to prepare infusions is also important. For example, green and oolong tea (*Camellia sinensis*) infusions prepared with water having a high mineral concentration and pH>6 changed their sensory profiles and resulted in darker infusions, attributed to the oxidation of the catechins (Xu et al. 2017). While studies on *Camellia sinensis* tea suggest that water quality may also impact the sensory and physicochemical properties of honeybush tea infusions, no such studies exist for honeybush. Given the unique sensory profile and phenolic composition of honeybush (Joubert et al. 2019), such an investigation is merited.

The consumer's perception is important as it determines the success of the product on the market (Grunert 2005). To date, all studies on the sensory profile and phenolic composition were of honeybush infusions prepared with distilled or deionized water (Du Preez 2020; Erasmus et al. 2017; Robertson et al. 2018). However, typically the choice of water for tea preparation is left to the consumer and depends on what is available or preferred (Delpla et al. 2020; Nkosi et al. 2021). Some consumers prefer bottled water over tap water due to the taste and perceived risks associated with drinking tap water (Delpla et al. 2020; Geerts et al. 2020). In South Africa, a water‐scarce country, water quality varies substantially, as demonstrated by groundwater from different regions having a wide range of electrical conductivity values (Nkosi et al. 2021; Verlicchi and Grillini 2019). Electrical conductivity, an indicator of water quality, is determined by the concentration of ions from dissolved salts and inorganic materials. According to South African standards, it should be 0‐170 mS/m, with an ideal range of 0‐70 mS/m, for drinkable water (DWAF 1996; SANS 2015).

The study hypothesis is that different water samples, varying in physicochemical properties such as pH and electrical conductivity, will affect the sensory and physicochemical properties of honeybush infusions. For this preliminary study, six water samples were used to brew infusions from three of the major commercialized *Cyclopia* species (i.e., *C. intermedia*, *C. subternata*, and *C. genistoides*). The experiments aimed to give a first indication of the water characteristics that are important for obtaining high‐quality infusions. These initial findings are intended to raise awareness among producers and tea merchants that water quality is an important consideration.

## 2 Materials and Methods

### 2.1 Chemicals

High‐performance liquid chromatography (HPLC)‐grade solvents were obtained from Merck (Darmstadt, Germany). Details of authentic reference standards for HPLC are given in Table S1 (Supplementary Information). Deionized water was prepared using an Elix water purification system and further purified to HPLC grade using a Milli‐Q academic water purification system from Merck. All other chemicals were from Merck or Sigma‐Aldrich (St. Louis, MO).

### 2.2 Honeybush and Water Samples

The sample set comprised 18 conventionally produced, including a high temperature oxidation step, honeybush herbal tea samples from *C. intermedia*, *C. subternata*, and *C. genistoides* (n = 6/species), selected from a large sample library. The samples comprised honeybush produced commercially (Agulhas Honeybush Tea, Bredasdorp, South Africa; Melmont Honeybush Tea, Kareedouw, South Africa; Cape Honeybush Tea Company, Mossel Bay, South Africa; Honeybush Natural Products, Clanwilliam, South Africa; Coetzee and Coetzee, Cape Town, South Africa) or on a laboratory scale (Agricultural Research Council, Stellenbosch, South Africa) spanning different production years. The samples were specifically selected to represent good quality honeybush tea.

Six water samples differing in pH and mineral composition were sourced for the preparation of the infusions: W1 (deionized water), W2 (bottled still spring water from Ceres, South Africa), W3 (bottled still spring water from Normandien, South Africa), W4 (bottled reverse osmosis water from Oasis, Stellenbosch, South Africa), W5 (brackish borehole water from a farm in Greyton, South Africa), and W6 (tap water from Stellenbosch, South Africa). W2, W3, and W4 are commercially available across South Africa from local retailers.

### 2.3 Physicochemical Analysis of Water Samples

The electrical conductivity and pH of the water samples were determined in duplicate using conductivity (Hanna Instruments Inc., Woonsocket, RI) and pH meters (Crison Instruments, Barcelona, Spain), respectively.

The anion content of the water samples was analyzed in triplicate using a Compact IC Flex oven/SES/PP/DEG (Metrohm, Herisau, Switzerland). A Metrosep A Supp 5 column (4.0 × 100 mm) connected to an A Supp 5 guard column was used for ion separation. Before analysis, 15 mL of each sample was filtered using a 0.45 µm nylon syringe filter. All samples were diluted using ultrapure water on a Metrohm 858 autosampler to a conductivity < 1000 µS/cm before injection, followed by prefiltering using an inline autofiltration cell (0.22 µm, cellulose). The mobile phase was 3 mM Na_2_CO_3_/2 mM NaHCO_3_ (1:1 ratio) with separation occurring at a flow rate of 0.8 mL/min and a column temperature of 30°C. Detection was performed using a conductivity detector (IC Professional Detector, Metrohm). Standard curves of all anions of interest were prepared. Data acquisition was performed using MagIC Net 3.3 software (Metrohm).

The total alkalinity was measured in duplicate using the BluVision Discrete analyzer (Skalar Analytical B.V., Breda, the Netherlands), and data collection and calculation were performed using the DiscreteAccess software. Each water sample was analyzed according to the method described in The Determination of Alkalinity and Acidity in Water 1981 (1982). Since the pH of the water samples was between 5.3 and 8.3, the total alkalinity was equal to the total bicarbonate (HCO_3_^−^) alkalinity and expressed as mg CaCO_3_/L.

For the detection of other elements at high to mid ppm levels and sub to mid ppm levels, inductively coupled plasma optical emission spectrometry (ICP‐OES) and ICP‐mass spectrometry (ICP‐MS) were used, respectively. These analyses were performed in duplicate using a Thermo iCAP 6000 series ICP‐OES (Thermo Scientific Inc., Waltham, MA) and an Agilent 7900 ICP‐MS (Agilent Technologies, Santa Clara, CA). The liquid samples were acidified to 2% final acid concentration using ultra‐pure HNO_3_ before analysis. For both instruments, the instrumental conditions are listed in the Supplementary Information (Table S2).

### 2.4 Preparation of Infusions

Infusions for sensory analysis were prepared at ‘cup‐of‐tea' strength according to Erasmus et al. (2017) directly before descriptive sensory analysis (DSA). Briefly, freshly boiled water (1 kg) was poured over the leaves (12.5 g), infused for 5 min, and strained through a fine‐mesh stainless‐steel strainer into a pre‐heated stainless‐steel flask. Infusions were served to the DSA panel in preheated white porcelain mugs labelled with three‐digit codes. All mugs were covered with a plastic lid and kept in temperature‐controlled (65°C) water baths. An unfiltered aliquot (ca. 30 mL) of each infusion was used for turbidity analysis, while another aliquot (ca. 100 mL) was filtered (Whatman No. 4 paper, GE Healthcare, Little Chalfont, UK) for color, soluble solids (SS), and HPLC analyses.

### 2.5 Descriptive Sensory Analysis (DSA)

DSA of the samples was conducted by a panel (n = 8) with extensive experience in the sensory analysis of honeybush tea (Du Preez 2020; Erasmus et al. 2017). Panelists were recruited through advertisements in a local newspaper and word of mouth. Those interested attended an introductory training session, after which they were screened for sensory acuity (Lawless and Heymann 2010). Participants who correctly identified at least 80% of the tests presented were selected to join the trained panel. During the training phase, panelists first evaluated samples individually. Then, the panel leader facilitated a discussion in which attributes were scored, feedback was provided, and a consensus was reached. The assessors were trained using the consensus and ballot method (Lawless and Heymann 2010) over four days (2 sessions/day). The panel was deemed sufficiently trained when they were able to consistently identify and score the intensities of attributes, thereby achieving a high level of agreement in their sensory assessment. Panel performance was monitored using Panelcheck software (Version 1.4.2, Nofima, Ås, Norway) (Tomic et al. 2007). Previously defined attributes (Du Preez 2020; Erasmus et al. 2017) were used for the sensory profiling of the honeybush infusions. These include attributes associated with all *Cyclopia* species (i.e., generic aroma and flavor attributes [“woody,” “fynbos‐floral,” “fynbos‐sweet,” “fruity‐sweet,” “apricot,” “raisin,” and “hay/dried grass”]). Species‐specific attributes include “rose perfume” (*C. intermedia*), “sweet spice” (*C. intermedia* and *C. subternata*), and “rose geranium” and bitter taste (*C. genistoides*) (Du Preez 2020; Robertson et al. 2018). Additional attributes identified during training, namely “date pudding” aroma and flavor, salty taste, and thick mouthfeel, were added (Table S3). During testing (3 days, 2 sessions/day for each *Cyclopia* species separately), infusions of one honeybush sample prepared using the six water samples were analyzed in one session in a random order for each assessor. The duration of each test session was approximately 50 min with a 10 min break between sessions. The intensity of each attribute was scored on unstructured line scales (0‐100) using Compusense five (Guelph, Canada). DSA was conducted in a light‐controlled room at 21°C. Filtered water and unflavored water biscuits were served as palate cleansers.

### 2.6 Physicochemical Properties of Honeybush Infusions

The CIEL*a*b* color values of the filtered infusions were measured in triplicate in 10‐mm path‐length polystyrene cuvettes, using the transmittance mode of a CM‐5 spectrophotometer (Konica Minolta Sensing Inc., Tokyo, Japan). The hue angle (h°) and chroma values were calculated by the instrument's software. Black and white standards were used for calibration. Turbidity of the unfiltered aliquots was determined in triplicate using a Hach 2100N IS Turbidimeter (Loveland, USA) and expressed in Nephelometric Turbidity Units (NTU). Calibration was performed using formazin standards. The soluble solids (SS) concentration (g/L) of the filtered infusions was determined gravimetrically (Joubert et al. 2022) using 20 mL aliquots.

The phenolic composition of the *C. intermedia* (De Beer et al. 2021)*, C. subternata* (De Beer et al. 2012), and *C. genistoides* (Beelders et al. 2014) infusions were quantified in duplicate using an Agilent 1200 HPLC with diode array detection. Aliquots of *C. intermedia* infusions (3 mL) with 100 µL of 10% ascorbic acid (w/v) added were evaporated under vacuum (5 h at 45°C and 5 mTorr; SpeedVac, ThermoFisher Scientific), and the residue was dissolved in 1 mL of deionized water. This was necessary to obtain peak areas suitable for quantification. Other infusions (1 mL) were only mixed with 100 µL of 10% ascorbic acid (w/v). All samples were filtered through 0.22 µm hydrophilic PVDF filters (Merck). Quantification of selected compounds was performed using external calibration (Supplementary Information, Table S3).

### 2.7 Statistical Analysis

The data for the infusions of the different *Cyclopia* species were analyzed separately. Each set of DSA data was pre‐processed to assess panel reliability by subjecting the data to a model that includes assessor, replicate, and sample effects and their interactions (Næs et al. 2010). Residuals were tested for non‐normality using the Shapiro‐Wilk test to identify outliers (*p* < 0.05), which were removed. Statistical analyses of DSA data were conducted on means over assessors per species, with honeybush samples (six for each species) as block replication and water samples as the main factor. Data for attributes with intensities less than 5 (100‐point intensity scale) for all infusions of a specific species were removed before data analysis, as they were deemed imperceptible (Erasmus et al. 2017) and did not have a large effect on the clustering of the samples (Figures S1–3, Supplementary Information). DSA and physicochemical data were subjected to analysis of variance (ANOVA) to test for treatment differences. Fisher's least significant difference was calculated to compare treatment means, with *p* < 0.05 considered significant (SAS statistical software, Version 9.4; SAS Institute, Cary, NC). Principal component analysis (PCA), using the correlation matrix, was conducted to visualize the effect of the water sample on the sensory profile of honeybush infusions (Lumivero 2024). Additional PCAs were conducted on the means of DSA data, with the mineral composition of the water samples included as supplementary variables (Figures S4‐6, Supplementary Information).

## 3 Results

### 3.1 Physicochemical Properties of Water Samples

Table 1 summarizes the values for electrical conductivity, pH, and concentration of anions and elements of each water sample. The pH ranged from slightly acidic (5.31; W2) to slightly alkaline (8.26; W3). W5 had the highest electrical conductivity (81 mS/m), while W1 and W4 had no measurable electrical conductivity. The total alkalinity was the highest for W3 (145.9 mg CaCO_3_/L), and the lowest for W1 and W4 (3.4 mg CaCO_3_/L). W1 and W4 also had the lowest concentration of anions and other elements. The Ca (34.12 mg/L), Ba (184.37 µg/L), and Sr (622.88 µg/L) ion concentrations were highest in W3, Na (137.2 mg/L), Mg (24.75 mg/L), B (44.37 µg/L), Mn (928.83 µg/L), Fe (335.52 µg/L), and Cu (33.41 µg/L) ion concentrations were highest in W5, and Cl^−^ (274.13 mg/L) and SO_4_^2−^ (49.54 mg/L) concentrations were highest in W6. W3 contained slightly less SO_4_^2−^ (41.06 mg/L) than W6, but it was undetectable in the other water samples.

**TABLE 1:** Physicochemical properties of water samples from different sources.

Parameter	Unit	Limit ^a^	LOQ	Water 1	Water 2	Water 3	Water 4	Water 5	Water 6
Electrical conductivity^b^	mS/m	≤170^c^		0 ± 0	1 ± 0	29 ± 0	0 ± 0	81 ± 0	4 ± 0
pH^b^		5–9.7^d^		7.05 ± 0.01	5.31 ± 0.04	8.26 ± 0.01	7.68 ± 0.01	6.54 ± 0.18	7.81 ± 0.01
Total alkalinity (HCO_3_^−^)^b^	mg CaCO_3_/L			3.4 ± 0.1	4.1 ± 0.1	145.9 ± 0.6	3.4 ± 0.1	22.5 ± 1.8	7.7 ± 0.1
Anions									
Bromide (Br^−^)	mg/L		0.05	<LOQ	<LOQ	<LOQ	<LOQ	<LOQ	0.90
Chloride (Cl^−^)	mg/L	≤300^c^	0.25	<LOQ	6.99	2.34	<LOQ	12.79	274.13
Fluoride (F^−^)	mg/L	≤1.5^e^	0.05	<LOQ	<LOQ	0.16	<LOQ	<LOQ	0.15
Nitrate (NO_3_^−^)	mg/L	≤11^e^	0.05	<LOQ	0.39	0.69	0.34	0.14	0.28
Nitrite (NO_2_^−^)	mg/L	≤0.9^e^	0.05	<LOQ	<LOQ	<LOQ	<LOQ	<LOQ	<LOQ
Phosphate (PO_4_^3−^)	mg/L		0.10	<LOQ	<LOQ	<LOQ	<LOQ	<LOQ	<LOQ
Sulphate (SO_4_^2−^)	mg/L	≤250^c^; ≤500^e^	1.50	<LOQ	<LOQ	41.06	<LOQ	<LOQ	49.54
Other elements									
*Major*									
Calcium (Ca)	mg/L		0.10	<LOQ	0.39	34.12	0.16	9.77	2.30
Magnesium (Mg)	mg/L		0.10	<LOQ	0.54	7.84	<LOQ	24.75	0.98
Phosphorus (P)	mg/L		0.10	<LOQ	<LOQ	<LOQ	<LOQ	<LOQ	<LOQ
Potassium (K)	mg/L		0.10	<LOQ	0.19	1.15	<LOQ	5.34	0.53
Silicon (Si)	mg/L		0.10	<LOQ	2.98	14.22	0.31	5.79	3.35
Sodium (Na)	mg/L	≤200^c^	0.10	<LOQ	4.013	41.67	0.48	137.2	6.65
*Minor*									
Aluminum (Al)	µg/L	≤300^d^	1.65	<LOQ	19.33	2.25	<LOQ	11.52	18.63
Antimony (Sb)	µg/L	≤20^e^	0.34	<LOQ	<LOQ	<LOQ	<LOQ	<LOQ	<LOQ
Arsenic (As)	µg/L	≤10^e^	0.04	<LOQ	<LOQ	2.49	<LOQ	0.30	0.09
Barium (Ba)	µg/L	≤700^e^	0.02	0.086	1.36	184.37	0.13	132.80	3.62
Boron (B)	µg/L	≤2400^e^	1.40	<LOQ	6.18	28.94	5.06	44.37	5.35
Cadmium (Cd)	µg/L	≤3^e^	0.003	0.007	0.009	<LOQ	<LOQ	0.13	0.01
Chromium (Cr)	µg/L	≤50^e^	0.11	<LOQ	0.127	<LOQ	<LOQ	<LOQ	0.13
Cobalt (Co)	µg/L		0.02	0.055	0.09	0.03	<LOQ	22.60	0.05
Copper (Cu)	µg/L	≤2000^e^	0.21	<LOQ	<LOQ	<LOQ	1.76	33.41	25.79
Iron (Fe)	µg/L	≤300^c^; ≤2000^e^	0.29	<LOQ	0.541	<LOQ	<LOQ	335.52	44.98
Lead (Pb)	µg/L	≤10^e^	0.02	<LOQ	<LOQ	<LOQ	<LOQ	0.30	0.26
Manganese (Mn)	µg/L	≤100^c^; ≤400^e^	0.04	0.11	1.23	0.16	0.072	928.83	2.09
Mercury (Hg)	µg/L	≤6^e^	0.10	<LOQ	<LOQ	<LOQ	<LOQ	<LOQ	<LOQ
Molybdenum (Mo)	µg/L		0.23	<LOQ	<LOQ	1.16	<LOQ	<LOQ	<LOQ
Nickel (Ni)	µg/L	≤70^e^	0.10	<LOQ	0.15	0.13	<LOQ	4.82	0.21
Selenium (Se)	µg/L	≤40^e^	0.11	<LOQ	<LOQ	0.081	<LOQ	0.12	<LOQ
Strontium (Sr)	µg/L		0.01	0.047	4.72	622.88	0.27	108.63	11.43
Tin (Sn)	µg/L		0.12	<LOQ	<LOQ	<LOQ	<LOQ	<LOQ	<LOQ
Vanadium (V)	µg/L		0.03	<LOQ	<LOQ	<LOQ	<LOQ	0.07	0.07
Zinc (Zn)	µg/L	≤5000^c^	0.14	<LOQ	0.380	<LOQ	1.80	36.27	15.05

*Note*: ^a^ SANS (2015); ^b^ mean ± standard deviation of duplicate measurements; ^c^ aesthetic risk; ^d^ operational risk; ^e^ acute or chronic health risk.

**Abbreviations**: LOQ, limit of quantification; W1, deionized water; W2, bottled still spring water from Ceres, South Africa; W3, bottled still spring water from Normandien, South Africa; W4, bottled reverse osmosis water from Oasis, Stellenbosch, South Africa; W5, brackish borehole water from a farm in Greyton, South Africa; W6, tap water from Stellenbosch, South Africa.

### 3.2 Sensory Profiles of Honeybush Infusions

**FIGURE 1:**
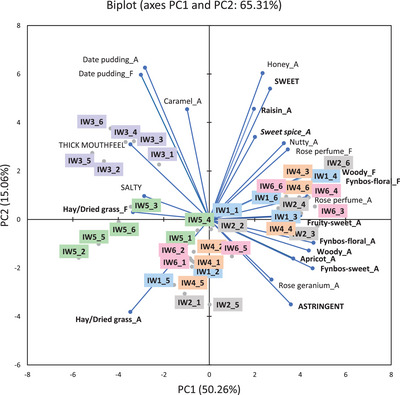
Principal component analysis bi‐plot representing the association between selected sensory attributes (attributes where all values <5 were removed) and “cup‐of‐tea” *Cyclopia intermedia* infusions prepared with water from different sources (n = 6). Sample labels indicate *C. intermedia* (I) infusion, followed by the water sample used to prepare the infusion (W1–W6), and lastly the *C. intermedia* sample number (IW1_1 = infusion prepared with W1 and *C. intermedia* sample 1). A indicates aroma attribute and F indicates flavor attribute. Attributes in **bold** font are generic honeybush sensory attributes, while those in ***italic bold*** font are defining characteristics of the specific species. W1, deionized water; W2, bottled still spring water from Ceres, South Africa; W3, bottled still spring water from Normandien, South Africa; W4, bottled reverse osmosis water from Oasis, Stellenbosch, South Africa; W5, brackish borehole water from a farm in Greyton, South Africa; W6, tap water from Stellenbosch, South Africa.

**FIGURE 2:**
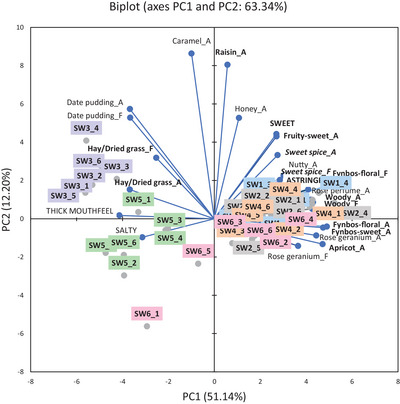
Principal component analysis bi‐plot representing the association between selected sensory attributes (attributes where all values <5 were removed) and "cup‐of‐tea" *Cyclopia subternata* infusions prepared with water from different sources (n = 6). Sample labels indicate *C. subternata* (S) infusion, followed by the water sample used to prepare the infusion (W1–W6), and lastly the *C. subternata* sample number (SW1_1 = infusion prepared with W1 and *C. subternata* sample 1). A indicates aroma attribute and F indicates flavor attribute. Attributes in **bold** font are generic honeybush sensory attributes, while those in ***italic bold*** font are defining characteristics of the specific species. W1, deionized water; W2, bottled still spring water from Ceres, South Africa; W3, bottled still spring water from Normandien, South Africa; W4, bottled reverse osmosis water from Oasis, Stellenbosch, South Africa; W5, brackish borehole water from a farm in Greyton, South Africa; W6, tap water from Stellenbosch, South Africa.

**FIGURE 3:**
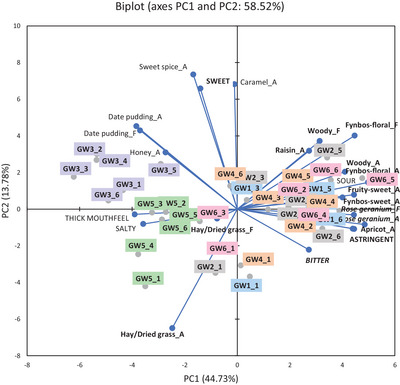
Principal component analysis bi‐plot representing the association between selected sensory attributes (attributes where all values <5 were removed) and "cup‐of‐tea" *Cyclopia genistoides* infusions prepared with water from different sources (n = 6). Sample labels indicate *C. genistoides* (G) infusion, followed by the water sample used to prepare the infusion (W1–W6), and lastly the *C. genistoides* sample number (GW1_1 = infusion prepared with W1 and *C. genistoides* sample 1). A indicates aroma attribute and F indicates flavor attribute. Attributes in **bold** font are generic honeybush sensory attributes, while those in ***italic bold*** font are defining characteristics of the specific species. W1, deionized water; W2, bottled still spring water from Ceres, South Africa; W3, bottled still spring water from Normandien, South Africa; W4, bottled reverse osmosis water from Oasis, Stellenbosch, South Africa; W5, brackish borehole water from a farm in Greyton, South Africa; W6, tap water from Stellenbosch, South Africa.

The results of infusions of *C. intermedia*, *C. subternata*, and *C. genistoides* prepared using six water samples were presented in PCA biplots (Figures 1, 2, 3), showing the overall trends per species with ANOVA results in Supplementary Information (Tables S4–S6). The following abbreviations were used to differentiate between samples: IWx, SWx, or GWx, where I = *C. intermedia*, S = *C. subternata*, and G = *C. genistoides*, and x referred to the number of the water sample (Table 1). Only data for the aroma, taste, and mouthfeel attributes were discussed in detail as flavor attributes, except “hay/dried grass,” was perceived at lower intensities than its corresponding aroma attribute and showed similar trends.

The PCA bi‐plots (Figures 1, 2, 3) for all three honeybush species showed the same general trends. Infusions brewed with W3 and W5 were distinctly separated from those made with other water samples on PC1. In general, infusions prepared with W3 and W5 (separated on PC2) were less associated with the typical generic honeybush aroma attributes. The intensity of the generic aroma attributes, such as “woody,” “fynbos‐floral,” “fynbos‐sweet,” “fruity‐sweet,” “apricot,” “raisin,” and “hay/dried grass,” was generally lower in infusions prepared with W3 and W5 (*p* < 0.05), with the lowest intensities observed for W3 (Tables S4–6). Infusions prepared from W3 and W5 were further separated by their association with the “date pudding” aroma attribute, thick mouthfeel, and salty taste. These attributes were significantly (*p* < 0.05) different from the infusions brewed with other water samples in most cases. Furthermore, the intensity of the “date pudding” aroma was considerably higher (*p* < 0.05) for W3 than for W5, whereas the salty taste and thick mouthfeel attributes were higher (*p* < 0.05) for W5. SW3 and IW3 were further separated due to their stronger association with the “hay/dried grass” aroma (Figure 2).

Although not clear from the PCAs (Figures 1, 2, 3), some significant differences were also observed in the defining aroma attributes for each species, as indicated by their intensities (Tables S4–S6). ‘Rose perfume’ aroma was lower (*p* < 0.05) in IW3, whereas “sweet spice” aroma was lower (*p* < 0.05) in IW5 compared to the other water samples. “Rose geranium” aroma was lower (*p* < 0.05) in GW3 and GW5 compared to the other water samples, with GW3 also being lower (*p* < 0.05) than GW5.

PCA bi‐plots for mean intensities of sensory attributes with the mineral concentrations included as supplementary variables, as well as correlation coefficients, indicated how the water composition parameters related to the sensory profile differences (Figures S1–3 and Table S7; Supplementary Information). For all the *Cyclopia* species, W3 infusions were associated with NO_3_^−^, SO_4_^2−^, F^−^, As, Sr, Ca, Si, and Ba. W1, W2, W4, and W6 infusions were associated with Cl^−^, Br^−^, Cr, Al, and Se, while W5 infusions were associated with the other anions and minerals.

### 3.3 Physicochemical Properties

The data for the color parameters, turbidity, and SS concentrations are presented in Table 2. The only differences (*p* < 0.05) in turbidity values compared to infusions prepared with deionized water (W1) were for IW5 (higher), GW3 (lower), and GW4 (lower).

**TABLE 2:** Images (100 mL per cup), color parameters (CIEL*a*b*), turbidity (NTU) and soluble solids content (g/L) of honeybush infusions prepared with water from different sources (mean ± standard deviation, n=6).

Species/ Parameter	W1	W2	W3	W4	W5	W6	*p*‐value
***Cyclopia intermedia***							
	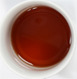	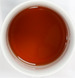	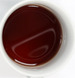	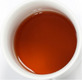	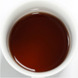	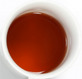	
L*	88.4a ± 7.7	88.5a ± 7.8	80.2c ± 8.8	88.7a ± 7.4	86.5b ± 8.3	88.3a ± 7.2	< 0.0001
a*	8.1b ± 5.7	7.9b ± 5.7	10.1a ± 7.8	7.8b ± 5.5	7.6b ± 6.2	7.8b ± 5.5	< 0.0001
b*	27.5b ± 17.7	27.1bc ± 17.7	38.7a ± 15.8	26.8bc ± 16.5	25.5c ± 16.7	27.0bc ± 16.3	< 0.0001
Chroma	28.6b ± 18.5	28.3b ± 18.5	41.6a ± 18.0	28.0b ± 17.4	26.6b ± 17.8	28.1b ± 17.2	< 0.0001
Hue angle	74.7c ± 3.4	74.9bc ± 3.6	70.7d ± 3.5	75.5ab ± 3.5	74.7c ± 4.6	75.9a ± 3.9	< 0.0001
Turbidity	12.9b ± 5.6	13.6b ± 8.9	9.1b ± 4.3	10.6b ± 8.1	25.1a ± 9.6	12.0b ± 10.6	< 0.0001
Soluble solids	1.23c ± 0.29	1.26c ± 0.28	1.43b ± 0.29	1.23c ± 0.29	1.70a ± 0.29	1.25c ± 0.27	< 0.0001
***Cyclopia subternata***							
	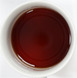	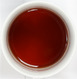	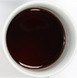	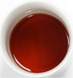	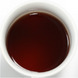	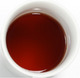	
L*	82.4ab ± 7.5	81.9ab ± 8.1	62.7d ± 3.3	81.5b ± 8.1	80.0c ± 7.8	82.7a ± 8.1	< 0.0001
a*	12.4bcd ± 6.3	12.8bc ± 6.7	22.8a ± 4.1	12.9b ± 6.7	11.7d ± 6.3	11.8 cd ± 7.0	< 0.0001
b*	35.5b ± 13.7	36.0b ± 14.1	54.1a ± 5.2	36.2b ± 13.8	34.4b ± 12.4	34.5b ± 14.4	< 0.0001
Chroma	37.7b ± 14.9	38.2b ± 15.4	59.2a ± 6.6	38.5b ± 15.1	36.4b ± 13.6	36.6b ± 15.7	< 0.0001
Hue angle	72.7abc ± 6.4	72.6bc ± 6.6	66.1d ± 2.2	72.4c ± 6.5	73.4a ± 7.2	73.3ab ± 6.9	< 0.0001
Turbidity	36.7a ± 25.2	35.0a ± 25.0	22.9a ± 16.6	31.8a ± 23.6	45.3a ± 21.3	41.1a ± 29.3	0.0802
Soluble solids	1.67d ± 0.39	1.76c ± 0.45	1.95b ± 0.43	1.71 cd ± 0.41	2.18a ± 0.39	1.67d ± 0.45	< 0.0001
***Cyclopia genistoides***							
	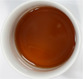	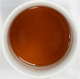	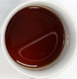	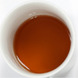	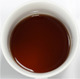	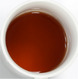	
L*	79.3b ± 6.3	80.1b ± 6.2	74.7d ± 1.7	77.6c ± 4.9	79.8b ± 5.0	81.3a ± 4.5	< 0.0001
a*	10.5 cd ± 5.3	9.9de ± 5.2	19.2a ± 3.3	12.4b ± 2.8	11.2c ± 5.5	9.4e ± 5.3	< 0.0001
b*	46.5 cd ± 9.8	45.7 cd ± 9.6	63.8a ± 2.5	50.4b ± 3.5	46.9c ± 9.8	45.5d ± 11.0	< 0.0001
Chroma	47.8 cd ± 10.6	46.9 cd ± 10.3	66.7a ± 3.3	51.9b ± 4.0	48.3c ± 10.6	46.5d ± 11.7	< 0.0001
Hue angle	78.3b ± 5.2	78.7b ± 5.3	73.3e ± 2.0	76.3d ± 2.3	77.6c ± 5.4	79.4a ± 5.6	< 0.0001
Turbidity	91.9a ± 56.5	77.1ab ± 35.2	62.7b ± 63.0	60.2b ± 18.3	76.5ab ± 43.0	88.3a ± 32.7	0.0002
Soluble solids	2.41c ± 0.23	2.36c ± 0.20	2.65b ± 0.20	2.40c ± 0.16	2.91a ± 0.29	2.39c ± 0.25	< 0.0001

*Note*: Different lowercase letters in a row indicate significant (*p* < 0.05) differences within a species. ANOVA was conducted with honeybush samples as block replication and water sample as the main factor. Fisher's least significant difference was calculated to compare treatment means, with *p* < 0.05 considered significant.

**Abbreviations**: NTU, Nephelometric Turbidity Units; W1, deionized water; W2, bottled still spring water from Ceres, South Africa; W3, bottled still spring water from Normandien, South Africa; W4, bottled reverse osmosis water from Oasis, Stellenbosch, South Africa; W5, brackish borehole water from a farm in Greyton, South Africa; W6, tap water from Stellenbosch, South Africa.

In terms of color parameters, W3 consistently stood out, having lower L* and h° and higher a*, b*, and chroma values (*p* < 0.05) compared to infusions prepared using the other water samples. W3 infusions also had higher SS concentrations (*p* < 0.05) compared to the corresponding W1 infusions. The trends for other water samples depend on the species, except for SS concentrations, which were higher (*p* < 0.05) for all W5 infusions compared to their corresponding W1 infusions: IW5 had lower L* and b* values (*p* < 0.05); SW5 and GW5 had lower L* and h° values (*p* < 0.05), respectively; IW2, GW4, and GW6 showed no significant differences (*p* ≥ 0.05) with their corresponding W1 infusions (Table 2); SW2 only differed from SW1 in that its SS concentration was higher (*p* < 0.05); IW4 and IW6 infusions had higher h° values (*p* < 0.05); and GW4 had higher a*, b*, and chroma values and lower L* and h° values (*p* < 0.05), while GW6 had lower a* values and higher L* and h° values (*p* < 0.05).

The type of brewing water significantly affected the concentration of the phenolic compounds, but the effect varied depending on the specific compound and the species (Table 3).

**TABLE 3:** Concentration (mg/L) of major phenolics in honeybush infusions prepared with water from different sources (mean ± standard deviation, n = 6).

Species/ Compound	W1	W2	W3	W4	W5	W6	*p*‐value
***Cyclopia intermedia***							
Unidentified	0.64a ± 0.34	0.63a ± 0.33	nd b	0.62a ± 0.32	0.45a ± 0.20	0.62a ± 0.31	< 0.0001
PTCA	2.53a ± 0.99	2.54a ± 1.11	1.27b ± 0.65	2.51a ± 1.08	2.41a ± 1.14	2.52a ± 1.10	< 0.0001
pCA	0.55a ± 0.21	0.53a ± 0.20	0.51a ± 0.19	0.53a ± 0.19	0.48a ± 0.17	0.53a ± 0.19	0.2843
IDG	5.53a ± 2.52	5.40a ± 2.42	4.94c ± 2.42	5.34ab ± 2.38	5.00bc ± 2.06	5.36a ± 2.37	0.0103
Mangiferin	4.23a ± 2.45	4.03a ± 2.33	1.52b ± 1.00	3.99a ± 2.12	3.44a ± 1.89	4.01a ± 2.17	< 0.0001
Isomangiferin	3.02a ± 1.60	2.92ab ± 1.49	1.34c ± 0.88	2.88ab ± 1.42	2.52b ± 1.27	2.89ab ± 1.44	< 0.0001
Vicenin‐2	2.93a ± 1.32	2.82a ± 1.23	2.85a ± 1.29	2.82a ± 1.22	2.55a ± 1.09	2.81a ± 1.21	0.2807
Eriocitrin	0.72a ± 0.72	0.73a ± 0.78	0.54a ± 0.58	0.71a ± 0.75	0.62a ± 0.73	0.69a ± 0.73	0.1744
Hesperidin	8.47a ± 1.76	8.02a ± 1.73	7.86a ± 1.76	8.35a ± 1.12	6.85a ± 3.08	8.22a ± 0.84	0.6094
Neoponcirin	0.76a ± 1.47	0.74a ± 1.37	0.71a ± 1.33	0.75a ± 1.42	0.70a ± 1.32	0.74a ± 1.42	0.5634
Hesperetin	0.64a ± 0.49	0.71a ± 0.58	0.22b ± 0.16	0.67a ± 0.57	0.55a ± 0.53	0.67a ± 0.55	0.0018
***Cyclopia subternata***							
PTCA	2.27a ± 0.51	2.38a ± 0.57	1.02b ± 0.77	2.43a ± 0.56	2.39a ± 0.60	2.44a ± 0.62	< 0.0001
pCA	0.47a ± 0.20	0.49a ± 0.22	0.42b ± 0.18	0.48a ± 0.20	0.47a ± 0.21	0.47a ± 0.24	0.0096
IDG	39.05ab ± 32.85	40.75a ± 35.20	35.37c ± 32.60	39.82ab ± 34.45	39.17ab ± 33.87	38.07b ± 37.91	0.0007
Mangiferin	1.66ab ± 1.52	1.83a ± 1.71	0.50c ± 0.72	1.75a ± 1.57	1.70ab ± 1.61	1.25b ± 0.98	< 0.0001
Isomangiferin	1.93ab ± 1.56	2.09a ± 1.76	0.95c ± 1.35	2.00ab ± 1.64	1.95ab ± 1.67	1.60b ± 1.49	< 0.0001
Vicenin‐2	2.61a ± 1.05	2.74a ± 1.22	2.72a ± 1.15	2.70a ± 1.14	2.62a ± 1.14	2.71a ± 1.27	0.1713
Scolymoside	8.41a ± 6.44	9.17a ± 7.26	7.01b ± 5.55	8.84a ± 6.83	8.67a ± 6.90	9.08a ± 7.85	0.0010
PDG	1.96a ± 1.07	2.02a ± 1.21	0.76c ± 0.83	1.99a ± 1.15	1.92a ± 1.13	1.63b ± 0.91	< 0.0001
Eriocitrin	3.94a ± 2.34	4.05a ± 2.50	2.63c ± 2.15	3.95a ± 2.29	3.88ab ± 2.34	3.46b ± 2.17	< 0.0001
Hesperidin	10.69a ± 6.53	10.78a ± 5.92	10.28a ± 6.31	11.06a ± 6.41	10.36a ± 5.39	11.53a ± 5.08	0.6059
***Cyclopia genistoides***							
MDH	1.31a ± 0.41	1.30a ± 0.40	0.98b ± 0.20	1.26a ± 0.36	1.34a ± 0.39	1.23a ± 0.19	0.0005
MMG	1.35a ± 1.04	1.32ab ± 1.00	0.35c ± 0.41	1.20ab ± 1.12	1.32a ± 0.99	0.97b ± 0.44	< 0.0001
IDG	40.62a ± 13.95	39.28a ± 12.80	39.76a ± 13.19	39.57a ± 13.42	39.38a ± 13.07	34.89a ± 6.12	0.2195
IMG	11.19a ± 10.43	10.84a ± 9.81	3.94c ± 4.45	10.61a ± 10.44	10.82a ± 9.80	7.03b ± 2.97	0.0001
Mangiferin	98.68a ± 38.29	93.72a ± 31.28	96.10a ± 42.64	95.78a ± 33.77	94.29a ± 31.19	98.76a ± 38.15	0.7553
Isomangiferin	33.57a ± 8.50	32.85a ± 7.32	29.42b ± 9.42	32.02a ± 7.65	31.89a ± 6.34	31.55ab ± 7.37	0.0114
Vicenin‐2	8.56a ± 1.76	8.26b ± 1.60	8.63a ± 1.56	8.38ab ± 1.64	8.25b ± 1.63	7.80c ± 0.75	0.0473
EDH	1.47a ± 0.47	1.43a ± 0.44	1.23b ± 0.43	1.43a ± 0.47	1.44a ± 0.45	1.43a ± 0.53	0.0002
2RNAR	4.62b ± 2.60	4.51bc ± 2.51	5.47a ± 2.54	4.60b ± 2.51	4.55b ± 2.36	4.17c ± 2.58	< 0.0001
2SNAR	5.92a ± 2.35	5.73ab ± 2.25	4.96c ± 2.13	5.80ab ± 2.40	5.65ab ± 2.22	5.52b ± 2.48	< 0.0001
Hesperidin	11.17bc ± 1.09	10.58c ± 1.46	12.61a ± 1.96	11.47b ± 1.89	11.08bc ± 1.99	11.74b ± 1.76	0.0009

*Note*: Different lowercase letters in a row indicate significant (*p* < 0.05) differences within a species. ANOVA was conducted with honeybush samples as block replication and water sample as the main factor. Fisher's least significant difference was calculated to compare treatment means, with *p* < 0.05 considered significant.

**Abbreviations**: 2RNAR, (2*R*)‐5‐*O*‐[α‐L‐rhamnopyranosyl‐(1→2)‐β‐D‐glucopyranosyl]naringenin; 2SNAR, (2*S*)‐5‐*O*‐[α‐L‐rhamnopyranosyl‐(1→2)‐β‐D‐glucopyranosyl]naringenin; EDH, eriodictyol‐O‐(deoxyhexose‐O‐hexose); IDG, 3‐β‐D‐glucopyranosyl‐4‐*O*‐β‐D‐glucopyranosyliriflophenone; IMG, 3‐β‐D‐glucopyranosyliriflophenone; MDH, maclurin‐di‐*O,C*‐hexoside; MMG, 3‐β‐D‐glucopyranosylmaclurin; nd, not detected (i.e., below limit of detection), values were set to zero for statistical analysis; pCA, *p*‐coumaric acid; PDG, 3',5'‐di‐β‐D‐glucopyranosylphloretin; PTCA, protocatechuic acid; W1, deionized water; W2, bottled still spring water from Ceres, South Africa; W3, bottled still spring water from Normandien, South Africa; W4, bottled reverse osmosis water from Oasis, Stellenbosch, South Africa; W5, brackish borehole water from a farm in Greyton, South Africa; W6, tap water from Stellenbosch, South Africa.

For *C. intermedia*, W3 produced infusions with lower concentrations of the unidentified compound, protocatechuic acid, 3‐β‐D‐glucopyranosyl‐4‐*O*‐β‐D‐glucopyranosyliriflophenone (IDG), hesperetin, mangiferin, and isomangiferin than W1 (*p* < 0.05) (Table 3). W5 resulted in lower concentrations of IDG, isomangiferin, and vicenin‐2 than IW1.

For *C. subternata*, SW3 had the lowest (*p* < 0.05) concentration of IDG, protocatechuic acid, eriocitrin, 3′,5′‐di‐β‐D‐glucopyranosylphloretin (PDG), mangiferin, isomangiferin, *p*‐coumaric acid, and scolymoside. SW6 showed a lower eriocitrin and 3′,5′‐di‐β‐D‐glucopyranosylphloretin (PDG) content than SW1, SW2, SW4, and SW5 (*p* < 0.05).

For *C. genistoides*, GW3 contained lower (*p* < 0.05) concentrations of maclurin‐di‐*O,C*‐hexoside (MDH), 3‐β‐D‐glucopyranosylmaclurin (MMG), IMG, eriodictyol‐*O*‐(deoxyhexose‐*O*‐hexose) (EDH), and (2*S*)‐5‐*O*‐[α‐L‐rhamnopyranosyl‐(1→2)‐β‐D‐glucopyranosyl]naringenin (2SNAR) than other infusions, but it had higher concentrations of hesperidin and (2*R*)‐5‐*O*‐[α‐L‐rhamnopyranosyl‐(1→2)‐β‐D‐glucopyranosyl]naringenin (2RNAR) (*p* < 0.05). GW6 had the lowest (*p* < 0.05) concentration of vicenin‐2 and 2RNAR, although 2RNAR was not different (*p* ≥ 0.05) from GW2. Isomangiferin was also lower in GW3, but not different (*p* ≥ 0.05) from GW6. The other water samples (W1, W2, W4, and W5) generally had a less pronounced effect on phenolic compounds in the *C. genistoides* infusions, with only slight differences in the concentration of specific compounds.

## 4 Discussion

### 4.1 Physicochemical Properties of Water Samples

The water samples for the study were selected to include options available to South African consumers. These comprised bottled spring water (W2 and W3), bottled reverse osmosis water (W4), brackish water (W5), and tap water (W6). Deionized water (W1), of high purity, served as the control and is typically used in the preparation of honeybush infusions for sensory profiling (Du Preez 2020; Erasmus et al. 2017; Robertson et al. 2018). Bottled water made up the majority of the water samples, as consumers are increasingly using bottled water due to the perceived risk associated with drinking tap water and its unacceptable taste (Delpla et al. 2020; Geerts et al. 2020). Brackish water was included in this study because many South Africans living on farms and in rural communities rely on borehole water (Nkosi et al. 2021). Given that the physicochemical properties of natural spring water and borehole water are subject to considerable variation, depending on the source and time of collection, the samples used in the present study were selected to represent different types of water to provide a preliminary indication of the potential impact of water quality on honeybush tea infusion quality.

The pH, electrical conductivity, and mineral content of the water samples were mostly within the limits set by the South African National Standards (SANS 2015) for drinking water. Exceptions were the chronic health and aesthetic risk levels of Mn (928.83 µg/L; limit ≤400 µg/L) and Fe (335.52 µg/L; limit ≤300 µg/L) ions, respectively, in W5, which can result in an unacceptable taste, as well as staining and encrustation of fixtures (DWAF 1996).

Considering the physicochemical properties of the water samples, W1 and W4 represent one end of the spectrum, and W3 and W5 represent the other. W1 and W4 had the lowest concentration of anions and cations. Deionized water has any electrically charged molecules and contaminants removed via an ion exchange process, while reverse osmosis removes dissolved constituents, including ions, organic compounds, and particulates (Bennett 2009). W3 and W5 had the highest concentration of the various anions and cations, confirmed by their high electrical conductivity. Treatment of tap water with chlorine explains the high Cl^−^ content of W6 (municipal tap water), a standard practice to control bacteria and odors, ensuring that the water is safe to drink (Sheibani and Mohammadi 2018).

### 4.2 Sensory Profile of Honeybush Infusions

The sensory profile of honeybush tea is a key quality characteristic, which, to date, has been investigated using deionized water (Joubert et al. 2019). This study examined the effect of brewing water on its sensory profile to guide consumers in selecting water for preparing the ideal cup of honeybush tea, similar to that brewed with deionized water. The ideal sensory profile has high intensities of positive/typical sensory attributes for a specific species and minimum intensities of negative or atypical sensory attributes.

The three *Cyclopia* species comprising the bulk of honeybush production were used to prepare infusions with different water samples. The generic and species‐specific sensory profiles of *C. intermedia*, *C. subternata*, and *C. genistoides* infusions were either not affected or only slightly affected when W2, W4, and W6 were used as brewing water. W3, and to a lesser extent, W5, had the most pronounced effect on the sensory profiles of the infusions for all the species.

The “date pudding” aroma note, not previously associated with honeybush tea, was observed at high intensity in W3 infusions. This aroma was described as “a sweet smell enhanced by the smell of bicarbonate of soda.” The high pH of W3 (8.26) may be responsible for the “date pudding” aroma, as a high pH could result in a soda taste (Sheibani and Mohammadi 2018). However, W4 and W6, with similar high pH levels (7–8) produced infusions with a barely perceptible “date pudding” aroma. The total alkalinity of W5 was the second highest (22.5 mg CaCO_3_/L), suggesting it may have contributed to the “date pudding” aroma, although it was much lower than that of W3 (145.3 mg CaCO_3_/L). W3 had both a high Na ion and HCO_3_^−^ concentration, which together with the high pH could have formed bicarbonate of soda (NaHCO_3_). Other notable differences in W3 were high Ba ion and SO_4_^2−^ content. Although a study on a medicinal preparation showed that Ba ions affect taste perception (Dietsch et al. 2014), the Ba ion level (40%) in that study was extreme compared to the level in W3 (184.37 µg/L). SO_4_^2−^ in water can give a salty, astringent, sweet, and bitter taste, depending on its association with Mg, Na, Zn, Fe, Ca, and Cu ions (Marcussen et al. 2013; Sheibani and Mohammadi 2018). It is thus postulated that the low‐intensity salty taste of W3 infusions could have been caused by SO_4_^2−^ forming salts with Na and Ca ions; however, the formation of these salts will have to be confirmed in future studies.

The lower perceived intensities of the generic honeybush aroma and palate attributes in W3 infusions could be due to suppression by the high intensity of the “date pudding” aroma, or the high pH and Ca ion concentrations of these water samples. Water with high pH (>7.3) and Ca ion concentration affected the taste of green tea by lowering the bitter, sweet, and umami tastes while increasing astringency (Xu et al. 2017; Yin et al. 2014). The conclusion was that water with a low pH (<6) and Ca ion concentration is necessary to preserve the characteristic taste of green tea. The high Ca ion concentration (>20 mg/L) in the brewing water inhibits the extraction of polyphenolic compounds present in green tea, thereby reducing the characteristic taste associated with these compounds (Yin et al. 2014). Another study on green tea by Sánchez‐López et al. (2020) found that the mineral content of the brewing water had a small but noticeable effect on the extraction of volatile organic compounds. The Ca ions present in the brewing water were absorbed by the tea leaf cells, modifying the diffusion of organic compounds from the leaves and resulting in a reduction of volatiles released. Future studies should investigate the volatile composition to determine if this is also true in the case of honeybush infusions.

Xu et al. (2017) showed that changing the pH of the brewing water changed the sensory profile of green, oolong, and black teas. However, for water with high electrical conductivity (>34 mS/m), the change in pH had a lesser effect on the sensory properties. Brewing water with a high electrical conductivity reduced the extraction of phenolic compounds, while a high pH (>7.9) affected their stability, subsequently affecting the sensory profile. When the electrical conductivity is high, more mineral ions are present in the water, which affects polyphenol stability through epimerization, oxidation, and/or degradation (Wang and Helliwell 2000). At an alkaline pH, polyphenols such as catechins have an increased proton‐donating potential that could lead to semiquinone free radical formation. Brewing water with a higher pH and electrical conductivity negatively affected the sensory profile of green tea, decreasing the characteristic bitter and umami tastes while increasing astringency and adding a salty taste (Cao et al. 2021; Franks et al. 2019). Cao et al. (2021) also found green tea to have an overall weaker flavor when prepared with water having a high pH and electrical conductivity.

In the present study, infusions prepared with W5 (high pH and electrical conductivity) also had lower perceived intensities of generic honeybush aroma and palate attributes. W5 had the highest concentrations of Fe (300 µg/L), Mg (24.8 mg/L), and Mn (900 µg/L) ions. The presence of Fe and Mg ions can cause bitter, metallic, astringent, salty, and sour tastes, depending on interactions with anions such as Cl^−^, SO_4_^2−^, and HCO_3_^−^ also present in water (Marcussen et al. 2013; Sheibani and Mohammadi 2018). It is also important to note that the oxidation of green tea polyphenols, such as catechins, can occur in the presence of Fe ions (>200 µg/L) (Wang and Helliwell 2000). It can be postulated that the high pH and electrical conductivity of W3 and W5, respectively, reduced the extraction of phenolic compounds that are likely responsible for the characteristic taste of honeybush infusions. High pH could also cause degradation of honeybush phenolics such as the xanthones mangiferin and isomangiferin and dihydrochalcones such as PDG. High pH negatively affects the stability of mangiferin (Beelders et al. 2018) and aspalathin (a dihydrochalcone) (De Beer et al. 2023) in solution.

New palate attributes, namely a thick mouthfeel and salty taste, were perceived for the infusions prepared with W5. The high salinity of W5 (brackish water) (Nkosi et al. 2021) likely caused the salty taste of the infusions. The high salinity of brackish water is related not only to the NaCl concentration but also to the concentration of various other ions, namely K, Ca, Mg, Zn, and Fe cations, as well as SO_4_^2−^, CO_3_^2−^, and NO_3_^−^, and their interaction with each other (Marcussen et al. 2013; Sheibani and Mohammadi 2018). Salts produced from Na^+^ associating with Cl^−^ and CO_3_^2−^ are saltier than those formed with HCO_3_^−^ and SO_4_^2−^ (Marcussen et al. 2013). Cao et al. (2021) reported that high Cl^−^ concentrations can impart a salty taste, depending on their association with Na, K, or Ca ions. W6 had the highest Cl^−^ concentration, but W5 had higher concentrations of Na, K, Ca, and Mg ions. Therefore, the Cl^−^ in W5, combined with Na, K, Ca, and/or Mg ions, could result in the formation of salts with a perceptible salty taste.

Astringency is defined as the puckering or drying sensation in the mouth caused by polyphenolic compounds present in tea (Zhang et al. 2020). Al, Ca, Cu, Fe, Mg, and Zn ions can also cause water to have an astringent taste, depending on their association with anions such as SO_4_^2−^, Cl^−^, and HCO_3_^−^ (Marcussen et al. 2013). Honeybush infusions are associated with a slight astringency (± 20 on the 100 point scale), contributing to the overall character of good quality honeybush tea (Du Preez et al. 2020). Infusions prepared with W3 and W5 were less astringent than those prepared with other water samples. Xu et al. (2017) found that green tea prepared with water having a high Ca ion concentration had increased astringency. It was proposed that the Ca ions promote the formation of complexes between epigallocatechin gallate (EGCG), a catechin responsible for the astringent and bitter taste in green tea, and proteins found in the oral cavity, causing the astringent sensation (Yin et al. 2014). Even though infusions prepared with W3 had the highest Ca ion concentration, an increase in astringency compared to the other infusions was not perceived. Research has shown that the electrical conductivity affects the extraction of polyphenols present in green tea, with higher electrical conductivity leading to less polyphenol extraction (Murugesh et al. 2017). The high electrical conductivity of W5 could have negatively affected the extraction of the phenolic compounds responsible for the astringency of honeybush tea, as well as inhibiting the binding of these polyphenols to the proteins in the mouth. A similar effect can be concluded for infusions prepared with W3. The phenolic compounds responsible for astringency in honeybush tea have not yet been identified, but several of the major phenolic compounds quantified in the infusions have structural features similar to compounds that cause astringency in *Camellia sinensis* teas. This includes phenolic compounds, having molecular masses higher than 500 amu and hydroxyl groups that can bind to the carbonyl group of the proteins through hydrogen bonding (Zhang et al. 2020).

Although the sensory profiles of infusions prepared with W6 were not significantly different from those prepared with W1, W2, and W4, its high Cl^−^ concentration could be detrimental to consumers’ health. Brewing tea with water that contains Cl^−^ (>19 mg/L) has the potential to form disinfection byproducts (DBP) (Li et al. 2021). DBPs form when Cl^−^ reacts with polyphenols, amino acids, caffeine, pigments, and/or aromatic compounds present in tea. While DBPs are already present in water with a high Cl^−^ content, new DBPs can also form when residual Cl^−^ reacts with compounds present in tea. Most DBPs, however, are removed during brewing due to their volatilization and adsorption onto the tea leaves (Li et al. 2021). The high Cl^−^ concentration (>200 mg/L) in W6 (tap water) could lead to the formation of DBPs with honeybush polyphenols. Therefore, honeybush tea prepared with water that has a high Cl^−^ should ideally be avoided.

### 4.3 Physicochemical Properties of Honeybush Infusions

The objective color measurements of the infusions were noticeably affected by the brewing water. Once again, W3 infusions stood out, having lower L* (darker) and h° values and higher a* (redness), b* (yellowness), and chroma values compared to the other infusions. The high chroma values indicate that the infusions had a more saturated color, and a darker shade of red‐brown, which was visually perceptible (Table 2). Franks et al. (2019) found that water with a higher pH and mineral concentration caused darker black and green tea infusions. At high pH (>7.4), hydroxyl groups of polyphenols dissociate, increasing their reactivity (Chobot et al. 2014). For example, at pH >6, the oxidation of tea catechins resulted in the formation of colored substances, darkening the infusion color (Xu et al. 2017). Cao et al. (2021) also showed that green, black, white, oolong, and dark tea colors darkened when brewed using water having a high pH (>7.4) and electrical conductivity. Concentrations of 0.935 mg/L for Ca ions and 0.092 mg/L for Mg ions were found to be the main contributors to this. The perceived dark color of infusions prepared with W3 could thus be the result of its high pH and concentration of mineral ions, such as Ca and Mg ions, affecting the stability of the phenolic compounds (Cao et al. 2021; Xu et al. 2017).

W5 also produced dark‐colored infusions, although to a lesser extent. These infusions had the highest SS content and turbidity values for *C. intermedia* and *C. subternata*. However, W1 and W6 produced *C. genistoides* infusions with the highest turbidity. The visually darker infusions prepared with W5 were confirmed for IW5 and SW5, having lower L* values compared to the infusions prepared with W1, W2, W4, and W6. Increased turbidity leads to a perceived darker infusion color due to the presence of more particles in the infusions (Gordillo et al. 2011). The high electrical conductivity of W5 could also have played a role in the turbidity of the infusions. The stability of green tea catechins in infusions was shown to be negatively affected by high electrical conductivity, resulting in their subsequent isomerization and degradation (Cao et al. 2021; Xu et al. 2017), as well as the formation of tea sediment, which increases the turbidity. Tea cream forms as tea cools due to polyphenolic compounds forming insoluble complexes through association with proteins, amino acids, and carbohydrates, which affect the color and taste of infusions (Murugesh et al. 2017). The presence of Ca and Mg ions in brewing water also increases the formation of tea cream in infusions due to complexes formed with phenolic compounds, which promote aggregation and precipitation (Franks et al. 2019; Murugesh et al. 2017). Therefore, the high electrical conductivity of W5 could have affected the stability of phenolic compounds by the minerals forming complexes with phenolic compounds, leading to the formation of insoluble compounds.

The turbidity values of all the *C. genistoides* infusions were higher than those of *C. intermedia* and *C. subternata* infusions, but still within the inter‐quartile range for turbidity (24.3–91.5 NTU) of a large honeybush sample set (n = 585) (Du Preez 2020). Therefore, it can be postulated that *C. genistoides* plant material produces more turbid infusions, irrespective of the brewing water, due to the differences in the phenolic composition between the different *Cyclopia* species (Beelders et al. 2014; De Beer et al. 2012; De Beer et al. 2021).

Several parameters that affect honeybush tea quality (i.e. taste, astringency, color, and turbidity) are directly related to their phenolic composition (Alexander et al. 2019a, Alexander et al. 2021; Bergh 2014; Du Preez 2020). Honeybush polyphenols also attract consumer attention due to their health‐promoting properties (Joubert et al. 2019). It is thus important to understand the impact of water quality on the phenolic content of the infusions. W3 negatively affected the concentration of many of the phenolic compounds. IW3, SW3, and GW3 had lower concentrations of most of the major phenolic compounds, with only GW3 containing higher concentrations of hesperidin and 2RNAR than the other infusions. Studies on green tea demonstrated that water with a high pH and electrical conductivity (Cao et al. 2021; Xu et al. 2017) results in lower concentrations of its main polyphenolic compounds. The degradation of catechins is pH‐dependent, and a high pH leads to their degradation and isomerization, resulting in lower concentrations in the final infusion (Wang and Helliwell 2000). It is postulated that the high pH and electrical conductivity of W3 caused some of the major *Cyclopia* phenolics to be unstable, resulting in their subsequent degradation. Alternatively, complexation of Ca, Mg, and Na ions with SO_4_^2−^ would also reduce solubility, leading to less extraction of the polyphenolic compounds from the leaves (Cao et al. 2021; Mossion et al. 2008; Xu et al. 2017; Yin et al. 2014).

Infusions prepared from *C. genistoides* were previously found to be bitter (Alexander et al. 2019a). In the present study, the *C. genistoides* infusions were also perceived as bitter. Studies by Alexander and co‐workers provide insight into phenolic compounds, which potentially contribute to or modulate the bitter taste of *C. genistoides* infusions (Alexander et al. 2019a, 2019b, Alexander et al. 2021). The GW3 and GW5 infusions were less bitter compared to the other *C. genistoides* infusions. Mangiferin is one of the compounds that contribute to the bitter taste of *C. genistoides* infusions (Alexander et al. 2019a), but its concentration was not affected by water type. Isomangiferin has been found to suppress the bitterness of mangiferin (Alexander et al. 2019a). In GW3, the isomangiferin concentration decreased (from 33 to 29 mg/L), but a decrease in perceived bitter taste was also perceived. The reduced bitterness could be due to the increased concentrations of hesperidin and 2RNAR since both compounds have bitter‐suppressing effects (Alexander et al. 2019b).

## 5 Conclusion

Different brewing waters significantly affected the sensory profile and physicochemical properties of honeybush infusions. The water samples with high pH and electrical conductivity proved detrimental to the sensory profile, color, and phenolic content of the infusions, providing a preliminary indication that these properties are important. These water samples not only reduced the generic and species‐specific sensory profiles of the infusions but also introduced new attributes uncharacteristic of honeybush, namely “date pudding” aroma and flavor, salty taste, and thick mouthfeel. Furthermore, all infusions prepared with these water samples were darker in color, despite having lower concentrations of most major phenolic compounds. While the tap water used in the present study did not substantially alter the sensory profile of honeybush tea, its high Cl^−^ concentration poses potential health risks due to the presence of unwanted disinfection byproducts. Our findings suggest that to achieve the characteristic sensory profile and red‐brown color of high quality honeybush tea, the ideal brewing water should have a slightly acidic to neutral pH and low electrical conductivity. In future work, elucidation of the separate effects of pH and electrical conductivity, as well as the individual minerals, would be beneficial for understanding the mechanisms involved and refining guidelines for brewing water. This can be achieved by using deionized water with adjusted pH and mineral content or a range of brackish water samples varying in these parameters.

## Nomenclature

2RNAR: (2*R*)‐5‐*O*‐[α‐L‐rhamnopyranosyl‐(1→2)‐β‐D‐glucopyranosyl]naringenin
2SNAR: (2*S*)‐5‐*O*‐[α‐L‐rhamnopyranosyl‐(1→2)‐β‐D‐glucopyranosyl]naringenin
ANOVA: analysis of variance
DBP: disinfection byproducts
DSA: descriptive sensory analysis
DWAF: Department of Water Affairs and Forestry
EC: electrical conductivity
EDH: eriodictyol‐*O*‐(deoxyhexose‐*O*‐hexose)
HPLC: high‐performance liquid chromatography
ICP OES: inductively coupled plasma optical emission spectrometry
ICP MS: inductively coupled plasma mass spectrometry
IDG: 3‐β‐D‐glucopyranosyl‐4‐*O*‐β‐D‐glucopyranosyliriflophenone
IMG: 3‐β‐D‐glucopyranosyliriflophenone
MDH: maclurin‐di‐*O,C*‐hexoside
MMG: 3‐β‐D‐glucopyranosylmaclurin
NTU: nephelometric turbidity units
PCA: principal component analysis
PDG: 3',5'‐di‐β‐D‐glucopyranosylphloretin
SANS: South African National Standards
SS: soluble solids.

## Author Contributions

**Helene van Schoor**: investigation. **Erika Moelich**: conceptualization, resources, supervision, writing – review and editing. **Brigitte von Pressentin du Preez**: visualization, writing – review and editing. **Chantelle Human**: writing – review and editing. **Dalene de Beer**: supervision, writing – original draft. **Elizabeth Joubert**: conceptualization, funding acquisition, writing – original draft, resources, supervision.

## Ethics Statement

This study was approved by the Research Ethics Committee for Social, Behavioral and Education Research (REC: SBE) at Stellenbosch University (project no. 9204). Participants provided written and informed consent prior to commencement of the study.

## Conflicts of Interest

The authors declare no conflicts of interest.

## Supporting information




**Supplementary Materials**: jfds70720‐sup‐0001‐SuppMat.pdf
