# Supplementary material for: The Sensory and Physicochemical Properties of Honeybush Tea Depend on the Brewing Water: A Preliminary Study
Source: J Food Sci. 2025 Nov 29;90(12):e70720. doi: 10.1111/1750-3841.70720 (PMC12663755; doi:10.1111/1750-3841.70720)
Supplement: Supplementary file 1 — Supplementary Materials: jfds70720‐sup‐0001‐SuppMat.pdf [file JFDS-90-0-s001.pdf]

SUPPLEMENTARY INFORMATION:

The sensory and physicochemical properties of honeybush tea depend on the brewing water: A preliminary study

Helene van Schoor[a,b], Erika Moelich[b], Brigitte von Pressentin du Preez[b], Chantelle Human[a], Dalene de Beer[a,b,*], Elizabeth Joubert[a,b]

[a] Plant Bioactives Group, Post-Harvest and Agro-Processing Technologies, Agricultural Research Council (Infruitec-Nietvoorbij), Stellenbosch, South Africa

[b] Department of Food Science, Stellenbosch University, Stellenbosch, South Africa

Correspondence: Dalene de Beer, Plant Bioactives Group, Post-Harvest and Agro-Processing Technologies, Agricultural Research Council (Infruitec-Nietvoorbij), Private Bag X5026, Stellenbosch 7599, South Africa. Email: dbeerd@arc.agric.za

Table S1 Details for quantification of phenolic compounds

Compound	Phenolic class	Wavelength (nm)	Quantification	Supplier
unknown	unknown	288	PTCA equivalents	-
protocatechuic acid (PTCA)	hydroxybenzoic acid	288	standard	Sigma (St Louis, USA)
p-coumaric acid (pCA)	hydroxycinnamic acid	320	standard	Sigma (St Louis, USA)
3-β-D-glucopyranosyl-4-*O*-β-D-glucopyranosyliriflophenone (IDG)	benzophenone	288	standard	isolated from *C. genistoides* [a]
maclurin-di-*O,C*-hexose (MDH)	benzophenone	288	MMG equivalents	-
3-β-D-glucopyranosylmaclurin (MMG)	benzophenone	288	standard	isolated from *C. genistoides* [a]
3-β-D-glucopyranosyliriflophenone (IMG)	benzophenone	288	standard	Phytolab (Vestenbergsgreuth, Germany)
mangiferin	xanthone	320	standard	Phytolab (Vestenbergsgreuth, Germany)
isomangiferin	xanthone	320	standard	Chemos (Regenstauf, Germany)
3′,5′-di-β-D-glucopyranosylphloretin (PDG)	dihydrochalcone	288	standard	isolated from *C. subternata* [b]
vicenin-2	flavone	320	standard	Phytolab (Vestenbergsgreuth, Germany)
scolymoside	flavone	320	standard	Sigma (St Louis, USA)
eriocitrin	flavanone	288	standard	Extrasynthese (Genay, France)
hesperidin	flavanone	288	standard	Sigma (St Louis, USA)
neoponcirin	flavanone	288	standard	Phytolab (Vestenbergsgreuth, Germany)
hesperetin	flavanone	288	standard	Sigma (St Louis, USA)
eriodictyol-*O*-(deoxyhexose-*O*-hexose) (EDH)	flavanone	288	eriocitrin equivalents	-
(2*R*)-5-*O*-[α-L-rhamnopyranosyl-(1→2)-β-D-glucopyranosyl]naringenin (2RNAR)	flavanone	288	standard	isolated from *C. genistoides* [c]
(2*S*)-5-*O*-[α-L-rhamnopyranosyl-(1→2)-β-D-glucopyranosyl]naringenin (2SNAR)	flavanone	288	standard	isolated from *C. genistoides* [c]

[a] Beelders, T., Brand, D. J., De Beer, D., Malherbe, C. J., Mazibuko, S. E., Muller, C. J. F., & Joubert, E. (2014). Benzophenone *C*- and *O*-glucosides from *Cyclopia genistoides* (honeybush) inhibit mammalian α-glucosidase. Journal of Natural Products, 77, 2694–2699. https://doi.org/10.1021/np5007247

[b] Human, C., Danton, O., De Beer, D., Maruyama, T., Alexander, L., Malherbe, C., Hamburger, M., & Joubert, E. (2021). Identification of a novel di-C-glycosyl dihydrochalcone and the thermal stability of polyphenols in model ready-to-drink beverage solutions with *Cyclopia subternata* extract as functional ingredient. Food Chemistry, 351, 129273. https://doi.org/10.1016/j.foodchem.2021.129273

[c] Danton, O., Alexander, L., Hunlun, C., De Beer, D., Hamburger, M., & Joubert, E. (2018). Bitter taste impact and thermal conversion of a naringenin glycoside from *Cyclopia genistoides*. Journal of Natural Products, 81, 2743–2749. https://doi.org/10.1021/acs.jnatprod.8b00710

Table S2 Instrumental conditions used for the detection of elements in water samples

**Thermo iCAP 6000series** [a]		**Agilent 7600** [b]	
Instrument component	Set level	Instrument component	Set level
RF power (W)	1350	RF power (W)	1600
Carrier gas (L/min) (Argon)	0.65	Carrier gas (L/min) (Argon)	0.83
Aux gas (L/min) (Argon)	1.0	Sample depth (mm)	10
Nebulizer	2 mL/min Micro mist	Nebulizer	0.4 mL/min Micro mist
Internal standard used	1 ppm Yttrium	Make-up gas (L/min)	0.15
		He flow (mL/min)	5
		H_2 flow (mL/min)	6

[a] Analysis of elements at high to mid ppm levels using inductively coupled plasma optical emission spectrometry (ICP-OES/AES) and iTEVA software (ThermoFisher Scientific, Waltham, USA).

[b] Analysis of elements at sub to mid ppm levels using inductively coupled plasma mass spectrometry (ICP-MS) and Masshunter software (Waters, Milford, USA).

Table S3 Description and reference standards for new aroma and palate attributes observed in infusions of the *Cyclopia* species prepared with water from different sources

	Description of attribute	***References standards***
**AROMA ATTRIBUTE**		
Date pudding	Aroma associated with date pudding baked with a high concentration of bicarbonate of soda. A sweet smell enhanced by the alkaline smell of bicarbonate of soda.	Mix 200 g pitted dates, 25 mL bicarbonate of soda and 311 g boiling water until well combined. Serve at room temperature.
**PALATE ATTRIBUTE**		
Date pudding	Flavor associated with date pudding with a high concentration of bicarbonate of soda. A sweet-associated flavor enhanced by the alkaline taste of bicarbonate of soda.	Mix 200 g pitted dates, 25 mL bicarbonate of soda and 311 g boiling water until well combined. Serve at room temperature.
Salty	A basic taste associated with a sodium chloride solution.	-
Thick mouthfeel	A physical sensation on the tongue and roof of the mouth, impression of a thick or slightly viscous liquid.	-

Table S4 Intensity scores for sensory attributes of *Cyclopia intermedia* infusions prepared with water from different sources (mean ± standard deviation)

Level	Water 1 (Deionized)	Water 2 (pH = 5)	Water 3 (pH = 8)	Water 4 (RO)	Water 5 (Brackish)	Water 6 (Tap)	p-value
Aroma							
**Woody**	43.2 ab ± 2.1	44.2 a ± 2.6	37.3 d ± 0.8	42.3 b ± 2.8	39.8 c ± 2.1	42.9 ab ± 2.5	<0.0001
**Fynbos-floral**	41.4 a ± 7.9	41.9 a ± 6.7	27.3 c ± 1.7	40.5 a ± 2.4	32.0 b ± 5.2	42.6 a ± 5.4	<0.0001
**Fynbos-sweet**	40.1 a ± 4.2	40.6 a ± 3.7	28.7 c ± 2.2	38.8 a ± 2.3	33.3 b ± 4.4	38.8 a ± 3.4	<0.0001
**Fruity-sweet**	26.7 a ± 3.8	26.8 a ± 3.1	22.1 c ± 1.7	26.0 ab ± 2.8	23.8 bc ± 3.5	27.8 a ± 2.4	0.0025
**Hay/dried grass**	18.1 a ± 2.0	17.9 a ± 3.5	19.6 a ± 0.5	18.7 a ± 2.7	19.6 a ± 0.6	17.8 a ± 2.7	0.1900
**Raisin**	27.5 a ± 3.3	27.5 a ± 3.8	27.8 a ± 3.7	27.9 a ± 3.1	27.4 a ± 2.5	29.2 a ± 3.3	0.9087
**Apricot**	13.0 ab ± 4.1	14.3 a ± 2.6	6.9 c ± 1.4	12.8 ab ± 5.0	10.2 b ± 3.3	14.6 a ± 2.9	0.0003
Rose perfume	13.1 a ± 5.4	15.3 a ± 5.6	7.8 c ± 3.2	12.8 ab ± 3.8	9.5 bc ± 5.1	13.4 a ± 4.6	0.0014
Sweet spice	7.3 a ± 1.5	7.5 a ± 2.5	8.0 a ± 1.6	8.0 a ± 1.5	4.3 b ± 1.6	7.2 a ± 1.2	0.0034
Caramel	10.7 b ± 1.6	9.9 b ± 2.9	13.5 a ± 1.9	11.7 ab ± 1.6	9.7 b ± 1.2	11.2 b ± 2.5	0.0118
Nutty	5.3 a ± 2.6	4.9 a ± 2.3	4.7 a ± 1.2	6.1 a ± 2.4	2.5 b ± 1.9	5.9 a ± 1.3	0.0010
Rose geranium	5.4 a ± 1.8	4.6 ab ± 2.1	2.7 c ± 0.9	4.9 a ± 1.6	2.9 bc ± 1.6	5.8 a ± 1.9	0.0069
Honey	6.4 a ± 2.4	5.9 a ± 2.2	6.9 a ± 1.4	5.5 a ± 2.4	4.5 a ± 2.2	6.1 a ± 1.6	0.1864
Date pudding	0.8 b ± 2.0	0.0 b ± 0.0	36.2 a ± 8.5	0.7 b ± 1.0	3.8 b ± 2.2	0.8 b ± 1.0	<0.0001
Flavor							
**Woody**	38.8 a ± 3.3	39.5 a ± 3.3	35.3 b ± 1.6	38.5 a ± 4.1	34.3 b ± 2.0	39.5 a ± 3.0	0.0001
**Fynbos-floral**	32.7 a ± 4.5	32.5 a ± 6.2	26.5 b ± 3.6	31.9 a ± 5.8	25.7 b ± 4.9	32.0 a ± 6.0	0.0012
**Hay/dried grass**	20.1 bc ± 1.2	18.3 d ± 1.8	22.3 a ± 1.2	19.7 cd ± 1.2	21.2 ab ± 1.5	19.5 cd ± 1.8	0.0001
Rose perfume	10.4 a ± 5.1	10.2 ab ± 6.6	7.0 bc ± 2.3	9.7 ab ± 5.3	5.6 c ± 3.8	8.9 abc ± 6.7	0.0395
Date pudding	0.9 bc ± 0.7	0.0 c ± 0.0	18.4 a ± 5.4	0.5 bc ± 0.7	3.0 b ± 1.4	0.3 bc ± 0.8	<0.0001
Taste and mouthfeel							
**SWEET**	21.5 a ± 1.7	21.6 a ± 2.0	22.0 a ± 1.6	21.0 a ± 2.1	20.4 a ± 1.2	21.7 a ± 1.7	0.0968
**ASTRINGENT**	24.7 a ± 1.0	25.5 a ± 1.3	20.1 b ± 0.9	25.7 a ± 0.5	19.1 b ± 1.7	25.4 a ± 1.1	<0.0001
SALTY	0.7 b ± 0.6	0.0 b ± 0.0	3.6 b ± 1.8	0.0 b ± 0.0	11.8 a ± 4.6	0.4 b ± 0.6	<0.0001
THICK MOUTHFEEL	0.6 c ± 0.7	0.2 c ± 0.5	11.7 b ± 3.0	0.2 c ± 0.5	15.5 a ± 3.5	0.5 c ± 0.7	<0.0001

Different lowercase letters in a row indicate significant ($p < 0.05$) differences. p-values indicate the significance of the water main effect.

Attributes in **bold font** are generic honeybush sensory attributes, while those in ***italic bold font*** are defining characteristics of the specific species.

Table S5 Intensity scores for sensory attributes of *Cyclopia subternata* infusions prepared with water from different sources (mean ± standard deviation)

Level	Water 1 (Deionized)	Water 2 (pH = 5)	Water 3 (pH = 8)	Water 4 (RO)	Water 5 (Brackish)	Water 6 (Tap)	p-values
Aroma							
**Woody**	45.4 a ± 0.7	45.1 a ± 1.6	39.2 c ± 1.5	45.5 a ± 1.7	41.4 bc ± 1.7	43.1 ab ± 4.2	0.0002
**Fynbos-floral**	42.7 ab ± 3.7	43.6 a ± 2.4	27.3 e ± 2.3	40.2 bc ± 2.1	31.7 d ± 3.3	39.5 c ± 3.7	<0.0001
**Fynbos-sweet**	40.3 a ± 1.5	39.9 a ± 1.8	27.7 d ± 1.4	39.8 ab ± 1.6	34.4 c ± 2.5	36.8 bc ± 5.1	<0.0001
**Fruity-sweet**	29.7 a ± 4.1	29.2 a ± 2.0	25.2 b ± 2.9	26.5 ab ± 2.1	25.7 b ± 2.3	25.0 b ± 4.7	0.0232
**Raisin**	29.2 a ± 1.6	28.8 a ± 2.2	30.4 a ± 3.9	29.1 a ± 2.2	27.2 a ± 2.3	26.2 a ± 4.0	0.1623
**Apricot**	22.7 a ± 3.3	21.7 a ± 1.2	7.7 c ± 1.3	22.1 a ± 1.6	14.8 b ± 1.4	19.7 a ± 5.3	<0.0001
**Hay/dried grass**	18.5 c ± 1.4	18.4 c ± 1.5	21.5 a ± 1.1	18.3 c ± 1.2	19.9 b ± 1.7	17.8 c ± 1.6	<0.0001
Sweet spice	10.8 a ± 1.7	11.0 a ± 1.1	8.5 b ± 1.3	9.7 ab ± 0.5	9.6 ab ± 1.2	8.4 b ± 2.0	0.0141
Rose geranium	10.3 a ± 3.1	10.3 a ± 3.1	2.5 c ± 1.3	9.9 a ± 1.8	6.2 b ± 1.3	9.1 a ± 2.6	<0.0001
Caramel	12.8 b ± 0.9	12.0 b ± 0.7	15.6 a ± 2.6	12.0 b ± 1.6	10.9 b ± 2.1	10.9 b ± 1.8	0.0012
Rose perfume	5.8 a ± 1.6	6.3 a ± 2.2	2.2 c ± 1.2	5.1 a ± 1.8	3.1 bc ± 0.7	4.6 ab ± 2.0	0.0014
Honey	6.0 a ± 1.8	5.4 a ± 1.4	5.5 a ± 1.8	6.3 a ± 1.0	6.2 a ± 2.1	4.3 a ± 3.5	0.2365
Date pudding	0.7 c ± 0.8	0.0 c ± 0.0	40.4 a ± 4.3	0.4 c ± 0.7	3.5 b ± 1.5	0.2 c ± 0.5	<0.0001
Nutty	7.3 ab ± 1.7	8.7 a ± 1.6	4.5 c ± 1.8	7.1 ab ± 3.1	6.2 bc ± 2.2	5.9 bc ± 3.0	0.0178
Flavor							
**Woody**	39.5 a ± 1.1	39.5 a ± 1.0	34.5 b ± 1.9	39.9 a ± 0.7	34.1 b ± 1.0	38.4 a ± 3.4	<0.0001
**Fynbos-floral**	32.9 a ± 2.4	32.8 a ± 3.4	22.8 c ± 1.1	31.4 ab ± 3.1	23.0 c ± 1.4	29.0 b ± 5.0	<0.0001
**Hay/dried grass**	19.4 b ± 1.0	20.1 b ± 1.5	21.8 a ± 2.0	19.9 b ± 1.1	21.6 a ± 1.3	19.6 b ± 1.7	0.0038
Sweet spice	6.0 a ± 1.5	5.8 ab ± 0.8	4.6 bc ± 1.3	6.0 a ± 1.0	4.0 c ± 1.2	5.1 abc ± 1.0	0.0164
Rose geranium	6.7 a ± 1.4	6.3 a ± 2.6	2.6 b ± 0.9	6.8 a ± 1.8	2.9 b ± 1.6	5.5 a ± 1.6	<0.0001
Date pudding	0.4 b ± 0.7	0.0 b ± 0.0	18.4 a ± 4.5	0.2 b ± 0.5	1.9 b ± 1.2	0.9 b ± 1.1	<0.0001
Taste and mouthfeel							
**SWEET**	22.0 a ± 1.2	21.4 ab ± 1.2	21.1 b ± 0.7	21.7 ab ± 0.9	19.6 c ± 0.9	21.2 b ± 0.9	<0.0001
**ASTRINGENT**	25.2 a ± 1.6	25.5 a ± 1.3	22.0 b ± 0.9	25.9 a ± 1.7	19.9 c ± 0.6	24.6 a ± 1.2	<0.0001
SALTY	0.3 c ± 0.8	0.4 c ± 0.7	3.9 b ± 0.6	0.0 c ± 0.0	11.4 a ± 2.9	0.1 c ± 0.3	<0.0001
THICK MOUTHFEEL	0.7 c ± 0.7	0.4 c ± 0.6	10.8 b ± 1.5	0.2 c ± 0.5	16.3 a ± 1.9	1.3 c ± 0.9	<0.0001

Different lowercase letters in a row indicate significant ($p < 0.05$) differences. p-values indicate the significance of the water main effect.

Attributes in **bold font** are generic honeybush sensory attributes, while those in ***italic bold font*** are defining characteristics of the specific species.

Table S6 Intensity scores for sensory attributes of *Cyclopia genistoides* infusions prepared with water from different sources (mean ± standard deviation)

Attribute	Water 1 (Deionized)	Water 2 (pH = 5)	Water 3 (pH = 8)	Water 4 (RO)	Water 5 (Brackish)	Water 6 (Tap)	p-value
Aroma							
**Woody**	42.1 a ± 1.8	42.5 a ± 1.2	38.1 b ± 3.0	41.9 a ± 1.7	38.9 b ± 1.4	41.9 a ± 2.7	0.0004
**Fynbos-floral**	39.5 a ± 4.5	39.0 ab ± 3.2	28.2 c ± 1.6	36.0 b ± 4.3	30.1 c ± 2.1	38.5 ab ± 4.7	<0.0001
**Fynbos-sweet**	36.0 a ± 2.3	35.3 a ± 2.1	30.7 b ± 2.2	35.1 a ± 1.6	32.0 b ± 2.1	34.8 a ± 2.2	<0.0001
**Fruity-sweet**	34.2 a ± 4.0	33.0 ab ± 6.0	25.5 c ± 4.2	32.3 ab ± 4.2	30.3 b ± 4.3	33.0 ab ± 7.1	0.0005
**Hay/dried grass**	19.9 a ± 1.1	19.4 a ± 2.2	20.1 a ± 1.7	19.8 a ± 1.3	21.3 a ± 1.6	19.7 a ± 1.5	0.2236
**Raisin**	28.2 a ± 1.8	27.3 a ± 2.6	27.0 a ± 3.1	29.6 a ± 2.6	25.5 a ± 3.9	28.2 a ± 3.4	0.0887
**Apricot**	28.4 a ± 7.3	27.9 a ± 6.7	11.6 c ± 2.8	28.1 a ± 8.7	19.9 b ± 4.6	27.5 a ± 7.6	<0.0001
Rose geranium	12.6 a ± 1.7	13.1 a ± 2.0	6.0 c ± 1.8	12.4 a ± 1.8	9.1 b ± 1.5	11.6 a ± 2.8	<0.0001
Caramel	12.1 bc ± 2.5	12 bc ± 2.9	14.8 a ± 1.8	12.8 b ± 1.9	10.7 c ± 2.0	12.0 bc ± 2.9	0.0040
Honey	4.1 b ± 2.0	3.1 b ± 1.7	7.8 a ± 2.4	3.5 b ± 2.1	3.7 b ± 2.2	2.9 b ± 1.8	0.0006
Date pudding	0.2 b ± 0.6	0.0 b ± 0.0	31.4 a ± 6.6	2.0 b ± 4.8	3.3 b ± 2.4	0.0 b ± 0.0	<0.0001
Sweet spice	2.7 b ± 1.6	3.4 b ± 1.7	6.1 a ± 2.0	3.2 b ± 1.5	2.9 b ± 1.9	3.9 b ± 1.7	0.0005
Flavor							
**Woody**	37.9 ab ± 2.5	36.3 c ± 1.3	35.0 c ± 1.0	36.4 bc ± 2.0	35.8 c ± 2.3	38.0 a ± 1.8	0.0045
**Fynbos-floral**	31.6 a ± 2.3	31.1 ab ± 2.2	26.5 c ± 0.5	29.6 b ± 2.4	26.2 c ± 1.8	30.8 ab ± 3.4	<0.0001
**Hay/dried grass**	21.8 a ± 2.0	21.0 a ± 0.6	22.7 a ± 0.9	21.8 a ± 1.2	21.8 a ± 3.0	21.7 a ± 1.6	0.5736
Rose geranium	10.2 a ± 2.1	8.1 b ± 3.3	3.5 d ± 1.9	9.5 ab ± 1.1	6.1 c ± 2.0	10.0 bc ± 2.8	<0.0001
Date pudding	0.0 b ± 0.0	0.0 b ± 0.0	15.6 a ± 5.3	0.6 b ± 1.4	1.0 b ± 0.9	0.4 b ± 1.0	<0.0001
Taste and mouthfeel							
**SWEET**	19.6 a ± 1.7	20.4 a ± 1.9	21.4 a ± 0.3	20.1 a ± 1.7	20.9 a ± 0.6	20.5 a ± 1.3	0.0931
**ASTRINGENT**	27.3 a ± 2.0	27.3 a ± 2.1	22.2 b ± 1.5	27.3 a ± 1.8	20.7 b ± 2.6	27.4 a ± 1.5	<0.0001
BITTER	10.3 a ± 4.0	9.7 ab ± 3.6	6.9 bc ± 1.8	8.8 ab ± 3.2	4.5 c ± 3.0	9.7 ab ± 3.7	0.0147
SALTY	0.0 c ± 0.0	0.0 c ± 0.0	4.4 b ± 0.8	0.5 c ± 0.8	9.4 a ± 4.5	0.1 c ± 0.3	<0.0001
THICK MOUTHFEEL	0.2 c ± 0.5	0.0 c ± 0.0	8.6 b ± 2.3	1.1 c ± 1.9	15.9 a ± 1.6	0.6 c ± 1.4	<0.0001

Different lowercase letters in a row indicate significant ($p < 0.05$) differences. p-values indicate the significance of the water main effect.

Attributes in **bold font** are generic honeybush sensory attributes, while those in ***italic bold font*** are defining characteristics of the specific species.

Table S7 Pearson's correlation (values in bold indicate $p < 0.05$) between mineral content and sensory attributes of *Cyclopia intermedia* infusions prepared with water from different sources.

Variables	Br^-	Cl^-	F^-	NO_3^{2-}	SO_4^{2-}	Ca	Mg	K	Si	Na	Al	As	Ba	B	Cd	Cr	Co	Cu	Fe	Pb	Mn	Ni	Se	Sr	V	Zn
Fynbos-floral_A	0.38	0.37	-0.37	-0.52	-0.23	**-0.92**	-0.68	-0.58	**-0.89**	-0.67	0.36	**-0.85**	**-0.99**	**-0.86**	-0.40	0.56	-0.43	-0.11	-0.39	-0.09	-0.43	-0.43	0.57	**-0.88**	-0.04	-0.26
Rose geranium_A	0.54	0.52	-0.12	-0.45	0.01	-0.79	-0.77	-0.68	-0.79	-0.76	0.24	-0.70	**-0.94**	**-0.91**	-0.54	0.53	-0.57	-0.13	-0.51	-0.09	-0.57	-0.56	0.39	-0.74	-0.03	-0.34
Rose perfume_A	0.25	0.25	-0.42	-0.39	-0.30	**-0.87**	-0.67	-0.58	-0.81	-0.66	0.47	-0.79	**-0.95**	**-0.82**	-0.40	0.64	-0.44	-0.21	-0.41	-0.19	-0.44	-0.44	0.52	**-0.83**	-0.15	-0.33
Apricot_A	0.44	0.43	-0.37	-0.54	-0.22	**-0.93**	-0.55	-0.44	**-0.87**	-0.54	0.53	**-0.89**	**-0.95**	-0.75	-0.25	0.65	-0.29	0.04	-0.24	0.07	-0.29	-0.29	0.67	**-0.91**	0.11	-0.11
Raisin_A	**0.96**	**0.96**	0.68	0.10	0.79	-0.09	-0.33	-0.28	-0.02	-0.32	0.39	-0.07	-0.25	-0.31	-0.31	0.61	-0.33	0.37	-0.21	0.42	-0.33	-0.31	-0.06	-0.10	0.50	0.06
Woody_A	0.25	0.24	-0.49	-0.53	-0.37	**-0.93**	-0.60	-0.50	**-0.88**	-0.59	0.43	**-0.87**	**-0.96**	-0.79	-0.31	0.57	-0.35	-0.14	-0.32	-0.12	-0.35	-0.35	0.62	**-0.89**	-0.08	-0.24
Fynbos-sweet_A	0.22	0.21	-0.52	-0.57	-0.40	**-0.95**	-0.62	-0.52	**-0.93**	-0.61	0.33	**-0.88**	**-0.98**	**-0.81**	-0.32	0.48	-0.36	-0.16	-0.33	-0.14	-0.36	-0.36	0.63	**-0.91**	-0.11	-0.26
Fruity-sweet_A	0.51	0.50	-0.26	-0.53	-0.11	**-0.89**	-0.64	-0.53	**-0.84**	-0.62	0.45	**-0.83**	**-0.96**	**-0.82**	-0.36	0.65	-0.40	0.00	-0.34	0.03	-0.40	-0.39	0.57	**-0.86**	0.09	-0.18
Honey_A	0.11	0.08	0.60	0.53	0.54	0.42	-0.62	-0.70	0.36	-0.63	-0.27	0.56	0.05	-0.41	-0.81	0.10	-0.80	-0.65	-0.80	-0.60	-0.80	-0.80	-0.81	0.50	-0.55	-0.78
Caramel_A	0.03	0.00	0.72	0.74	0.62	0.73	-0.24	-0.34	0.62	-0.25	-0.56	0.81	0.44	0.02	-0.52	-0.30	-0.49	-0.40	-0.49	-0.39	-0.49	-0.49	**-0.90**	0.77	-0.36	-0.47
Date pudding_A	-0.21	-0.22	0.66	0.79	0.52	**0.98**	0.19	0.07	**0.94**	0.18	-0.40	**1.00**	**0.83**	0.47	-0.15	-0.35	-0.11	-0.26	-0.14	-0.25	-0.11	-0.11	**-0.92**	**1.00**	-0.26	-0.21
Sweet spice_A	0.05	0.01	0.31	0.53	0.28	0.07	**-0.88**	**-0.93**	-0.01	**-0.89**	-0.29	0.24	-0.33	-0.71	**-0.98**	0.15	**-0.97**	**-0.82**	**-0.98**	-0.79	**-0.97**	**-0.97**	-0.60	0.17	-0.73	**-0.94**
Nutty_A	0.36	0.32	0.22	0.18	0.26	-0.31	**-0.94**	**-0.93**	-0.37	**-0.95**	-0.14	-0.15	-0.65	**-0.89**	**-0.91**	0.31	**-0.91**	-0.55	**-0.88**	-0.51	**-0.91**	**-0.91**	-0.23	-0.22	-0.44	-0.75
Hay/dried grass_A	-0.46	-0.45	0.13	0.33	0.01	0.74	0.77	0.69	0.69	0.76	-0.46	0.65	**0.90**	**0.88**	0.55	-0.72	0.59	0.21	0.54	0.16	0.59	0.58	-0.34	0.69	0.10	0.40
SWEET	0.28	0.25	0.65	0.56	0.62	0.32	-0.68	-0.74	0.32	-0.68	0.01	0.46	-0.06	-0.48	**-0.83**	0.37	**-0.84**	-0.57	**-0.82**	-0.51	**-0.84**	**-0.83**	-0.73	0.40	-0.44	-0.74
ASTRINGENT	0.32	0.30	-0.20	-0.20	-0.11	-0.74	**-0.88**	**-0.82**	-0.75	**-0.88**	0.20	-0.62	**-0.95**	**-0.96**	-0.68	0.52	-0.71	-0.39	-0.68	-0.37	-0.71	-0.71	0.27	-0.67	-0.30	-0.56
SALTY	-0.25	-0.21	-0.11	-0.18	-0.15	0.35	**1.00**	**0.99**	0.39	**1.00**	0.01	0.19	0.70	**0.94**	**0.95**	-0.42	**0.96**	0.67	**0.94**	0.63	**0.96**	**0.96**	0.21	0.26	0.56	**0.85**
THICK MOUTHFEEL	-0.31	-0.28	0.17	0.20	0.08	0.71	**0.92**	**0.86**	0.72	**0.91**	-0.15	0.57	**0.93**	**0.99**	0.73	-0.49	0.76	0.45	0.73	0.42	0.76	0.75	-0.20	0.63	0.36	0.62
Fynbos-floral_F	0.27	0.24	-0.27	-0.31	-0.17	-0.77	**-0.87**	-0.80	-0.78	**-0.86**	0.18	-0.65	**-0.96**	**-0.97**	-0.66	0.49	-0.68	-0.42	-0.66	-0.39	-0.68	-0.68	0.30	-0.70	-0.33	-0.57
Rose perfume_F	0.06	0.03	-0.30	-0.19	-0.25	-0.65	**-0.91**	**-0.87**	-0.69	**-0.91**	0.02	-0.51	**-0.88**	**-0.97**	-0.75	0.35	-0.76	-0.63	-0.77	-0.60	-0.76	-0.77	0.14	-0.56	-0.56	-0.73
Woody_F	0.40	0.38	-0.13	-0.21	-0.02	-0.72	**-0.88**	**-0.81**	-0.69	**-0.87**	0.32	-0.60	**-0.93**	**-0.96**	-0.69	0.63	-0.72	-0.36	-0.68	-0.32	-0.72	-0.71	0.24	-0.65	-0.25	-0.55
Date pudding_F	-0.24	-0.25	0.64	0.76	0.49	**0.99**	0.25	0.12	**0.94**	0.23	-0.42	**1.00**	**0.86**	0.51	-0.09	-0.39	-0.06	-0.22	-0.09	-0.22	-0.06	-0.06	**-0.90**	**1.00**	-0.23	-0.17
Hay/dried grass_F	-0.23	-0.22	0.44	0.32	0.33	**0.85**	0.59	0.50	0.77	0.58	-0.56	0.79	**0.90**	0.73	0.33	-0.67	0.36	0.16	0.34	0.14	0.36	0.36	-0.55	**0.81**	0.11	0.26

A indicates aroma attribute and F indicates flavor attribute

Table S8 Pearson's correlation (values in bold indicate $p < 0.05$) between mineral content and sensory attributes of *Cyclopia subternata* infusions prepared with water from different sources.

Variables	Br^-	Cl^-	F^-	NO_3^{2-}	SO_4^{2-}	Ca	Mg	K	Si	Na	Al	As	Ba	B	Cd	Cr	Co	Cu	Fe	Pb	Mn	Ni	Se	Sr	V	Zn
Fynbos-floral_A	0.15	0.14	-0.52	-0.51	-0.41	**-0.91**	-0.68	-0.59	**-0.89**	-0.67	0.29	**-0.82**	**-0.97**	**-0.85**	-0.40	0.46	-0.43	-0.28	-0.42	-0.26	-0.43	-0.43	0.55	**-0.86**	-0.23	-0.36
Rose geranium_A	0.16	0.15	-0.60	-0.62	-0.48	**-0.97**	-0.57	-0.46	**-0.96**	-0.55	0.27	**-0.92**	**-0.97**	-0.77	-0.26	0.39	-0.29	-0.14	-0.27	-0.13	-0.29	-0.29	0.69	**-0.94**	-0.10	-0.21
Rose perfume_A	0.03	0.02	-0.56	-0.45	-0.48	**-0.86**	-0.68	-0.61	**-0.85**	-0.67	0.25	-0.77	**-0.94**	**-0.84**	-0.42	0.42	-0.45	-0.37	-0.46	-0.36	-0.45	-0.45	0.50	-0.81	-0.33	-0.43
Apricot_A	0.13	0.12	-0.62	-0.65	-0.50	**-0.97**	-0.55	-0.45	**-0.98**	-0.54	0.20	**-0.92**	**-0.97**	-0.77	-0.25	0.33	-0.27	-0.14	-0.26	-0.14	-0.27	-0.28	0.69	**-0.94**	-0.11	-0.21
Raisin_A	-0.74	-0.76	-0.04	0.52	-0.20	0.47	-0.25	-0.37	0.34	-0.27	-0.69	0.57	0.28	-0.06	-0.45	-0.54	-0.42	**-0.87**	-0.52	**-0.89**	-0.42	-0.43	-0.68	0.55	**-0.91**	-0.72
Woody_A	-0.03	-0.04	-0.66	-0.55	-0.58	**-0.91**	-0.62	-0.54	**-0.94**	-0.61	0.07	**-0.84**	**-0.94**	-0.80	-0.35	0.22	-0.36	-0.33	-0.37	-0.33	-0.36	-0.37	0.58	**-0.86**	-0.31	-0.36
Fynbos-sweet_A	0.03	0.02	-0.71	-0.67	-0.60	**-0.97**	-0.49	-0.39	**-0.97**	-0.48	0.19	**-0.92**	**-0.93**	-0.71	-0.18	0.27	-0.21	-0.15	-0.21	-0.16	-0.21	-0.21	0.72	**-0.94**	-0.14	-0.18
Fruity-sweet_A	-0.46	-0.47	-0.67	-0.41	-0.68	-0.52	-0.42	-0.40	-0.55	-0.42	-0.05	-0.45	-0.54	-0.52	-0.26	0.01	-0.27	-0.56	-0.34	-0.57	-0.27	-0.28	0.27	-0.46	-0.58	-0.47
Honey_A	**-0.87**	**-0.87**	-0.71	-0.24	-0.79	0.00	0.35	0.32	-0.14	0.34	-0.66	-0.03	0.19	0.30	0.36	**-0.86**	0.39	-0.23	0.28	-0.30	0.39	0.38	0.17	-0.01	-0.38	0.05
Caramel_A	-0.40	-0.42	0.45	0.71	0.30	0.79	-0.16	-0.29	0.69	-0.18	-0.59	**0.87**	0.54	0.10	-0.46	-0.41	-0.42	-0.66	-0.49	-0.65	-0.42	-0.43	**-0.94**	**0.85**	-0.65	-0.60
Date pudding_A	-0.22	-0.23	0.66	0.80	0.52	**0.98**	0.18	0.06	**0.93**	0.17	-0.41	**1.00**	**0.82**	0.46	-0.16	-0.35	-0.12	-0.27	-0.15	-0.26	-0.12	-0.12	**-0.93**	**1.00**	-0.27	-0.23
Sweet-spice_A	-0.56	-0.56	**-0.85**	-0.47	**-0.85**	-0.57	-0.22	-0.19	-0.59	-0.21	0.01	-0.54	-0.50	-0.36	-0.03	-0.05	-0.04	-0.42	-0.12	-0.44	-0.04	-0.05	0.44	-0.54	-0.48	-0.27
Nutty_A	-0.24	-0.24	-0.78	-0.44	-0.72	-0.80	-0.39	-0.32	-0.76	-0.38	0.33	-0.76	-0.75	-0.55	-0.13	0.32	-0.15	-0.29	-0.19	-0.30	-0.15	-0.16	0.60	-0.77	-0.31	-0.24
Hay/dried grass_A	-0.44	-0.44	0.38	0.58	0.23	**0.95**	0.56	0.45	**0.91**	0.55	-0.40	**0.90**	**0.97**	0.77	0.26	-0.55	0.30	-0.05	0.24	-0.07	0.30	0.29	-0.68	**0.93**	-0.12	0.10
SWEET	-0.01	-0.05	-0.03	0.07	-0.03	-0.30	**-0.95**	**-0.96**	-0.40	**-0.96**	-0.30	-0.13	-0.63	**-0.90**	**-0.92**	0.11	**-0.92**	**-0.81**	**-0.94**	-0.78	**-0.92**	**-0.92**	-0.26	-0.19	-0.73	**-0.92**
ASTRINGENT	0.15	0.12	-0.20	-0.08	-0.14	-0.62	**-0.95**	**-0.91**	-0.66	**-0.94**	0.02	-0.47	**-0.87**	**-0.98**	-0.80	0.37	**-0.81**	-0.61	-0.81	-0.58	**-0.81**	**-0.82**	0.09	-0.53	-0.53	-0.74
SALTY	-0.28	-0.25	-0.10	-0.11	-0.15	0.40	**1.00**	**0.98**	0.44	**1.00**	0.01	0.23	0.73	**0.96**	**0.93**	-0.42	**0.94**	0.64	**0.92**	0.59	**0.94**	**0.94**	0.17	0.30	0.52	**0.82**
THICK MOUTHFEEL	-0.26	-0.23	0.15	0.13	0.07	0.65	**0.95**	**0.90**	0.67	**0.94**	-0.09	0.50	**0.90**	**0.99**	0.79	-0.45	0.81	0.53	0.79	0.50	0.81	0.81	-0.12	0.56	0.43	0.69
Fynbos-floral_F	0.04	0.02	-0.48	-0.38	-0.40	-0.80	-0.80	-0.73	**-0.83**	-0.79	0.10	-0.69	**-0.94**	**-0.92**	-0.57	0.35	-0.59	-0.49	-0.60	-0.48	-0.59	-0.59	0.37	-0.73	-0.44	-0.57
Rose geranium_F	0.11	0.08	-0.46	-0.41	-0.37	**-0.84**	-0.79	-0.72	**-0.88**	-0.78	0.04	-0.73	**-0.97**	**-0.92**	-0.55	0.30	-0.57	-0.42	-0.57	-0.41	-0.57	-0.57	0.41	-0.77	-0.37	-0.51
Woody_F	0.14	0.12	-0.38	-0.30	-0.30	-0.79	**-0.85**	-0.79	**-0.82**	**-0.84**	0.08	-0.67	**-0.96**	**-0.95**	-0.63	0.37	-0.65	-0.47	-0.64	-0.45	-0.65	-0.65	0.33	-0.72	-0.40	-0.58
Date pudding_F	-0.19	-0.19	0.68	0.79	0.55	**0.98**	0.19	0.06	**0.94**	0.17	-0.39	**1.00**	**0.82**	0.46	-0.15	-0.33	-0.12	-0.24	-0.15	-0.23	-0.12	-0.12	**-0.92**	**1.00**	-0.24	-0.21
Sweet spice_F	-0.07	-0.10	-0.38	-0.18	-0.34	-0.64	**-0.88**	**-0.85**	-0.71	**-0.88**	-0.13	-0.49	**-0.85**	**-0.93**	-0.73	0.18	-0.74	-0.68	-0.76	-0.67	-0.74	-0.74	0.13	-0.54	-0.63	-0.75
Hay/dried grass_F	-0.39	-0.37	0.26	0.48	0.14	**0.83**	0.79	0.70	**0.85**	0.78	-0.16	0.73	**0.97**	**0.93**	0.54	-0.44	0.57	0.24	0.53	0.20	0.57	0.57	-0.41	0.77	0.15	0.41

A indicates aroma attribute and F indicates flavor attribute

Table S9 Pearson's correlation (values in bold indicate $p < 0.05$) between mineral content and sensory attributes of *Cyclopia genistoides* infusions prepared with water from different sources.

Variables	Br^-	Cl^-	F^-	NO_3^{2-}	SO_4^{2-}	Ca	Mg	K	Si	Na	Al	As	Ba	B	Cd	Cr	Co	Cu	Fe	Pb	Mn	Ni	Se	Sr	V	Zn
Fynbos-floral_A	0.33	0.31	-0.34	-0.49	-0.22	**-0.85**	-0.73	-0.65	**-0.83**	-0.72	0.33	-0.77	**-0.97**	**-0.89**	-0.47	0.55	-0.51	-0.23	-0.48	-0.19	-0.51	-0.51	0.47	-0.81	-0.14	-0.37
Rose geranium_A	0.15	0.14	-0.60	-0.59	-0.48	**-0.96**	-0.58	-0.48	**-0.95**	-0.56	0.30	**-0.91**	**-0.97**	-0.78	-0.27	0.42	-0.30	-0.16	-0.29	-0.15	-0.30	-0.30	0.67	**-0.93**	-0.12	-0.23
Apricot_A	0.26	0.25	-0.53	-0.63	-0.40	**-0.98**	-0.56	-0.45	**-0.96**	-0.55	0.29	**-0.93**	**-0.98**	-0.78	-0.25	0.42	-0.28	-0.06	-0.25	-0.05	-0.28	-0.28	0.70	**-0.95**	-0.02	-0.16
Raisin_A	0.21	0.17	-0.02	0.00	0.03	-0.43	**-0.84**	**-0.81**	-0.54	**-0.84**	-0.31	-0.30	-0.68	**-0.83**	-0.76	0.08	-0.75	-0.50	-0.74	-0.48	-0.75	-0.76	-0.04	-0.35	-0.43	-0.64
Woody_A	0.26	0.24	-0.41	-0.44	-0.29	**-0.88**	-0.75	-0.66	**-0.87**	-0.74	0.29	-0.79	**-0.99**	**-0.90**	-0.49	0.51	-0.52	-0.27	-0.49	-0.25	-0.52	-0.52	0.49	**-0.83**	-0.21	-0.40
Fynbos-sweet_A	0.19	0.17	-0.48	-0.56	-0.38	**-0.90**	-0.71	-0.62	**-0.92**	-0.70	0.17	**-0.82**	**-0.98**	**-0.88**	-0.43	0.38	-0.46	-0.28	-0.45	-0.26	-0.46	-0.46	0.53	**-0.85**	-0.22	-0.38
Fruity-sweet_A	0.25	0.24	-0.57	-0.76	-0.44	**-0.98**	-0.45	-0.33	**-0.96**	-0.44	0.32	**-0.95**	**-0.93**	-0.69	-0.13	0.40	-0.16	0.03	-0.13	0.04	-0.16	-0.16	0.77	**-0.96**	0.07	-0.05
Honey_A	-0.33	-0.34	0.55	0.69	0.40	**0.95**	0.17	0.04	**0.86**	0.15	-0.56	**0.97**	0.80	0.43	-0.16	-0.50	-0.12	-0.35	-0.17	-0.34	-0.12	-0.13	**-0.91**	**0.97**	-0.36	-0.27
Caramel_A	-0.15	-0.18	0.60	**0.84**	0.49	0.70	-0.37	-0.48	0.61	-0.38	-0.50	**0.81**	0.38	-0.09	-0.66	-0.22	-0.62	-0.65	-0.65	-0.64	-0.62	-0.63	**-0.96**	0.77	-0.61	-0.69
Date pudding_A	-0.24	-0.25	0.64	0.81	0.50	**0.98**	0.19	0.07	**0.93**	0.18	-0.43	**1.00**	**0.82**	0.47	-0.15	-0.38	-0.11	-0.27	-0.15	-0.27	-0.11	-0.12	**-0.92**	**0.99**	-0.28	-0.22
Sweet spice_A	0.08	0.07	**0.84**	**0.91**	0.74	**0.88**	-0.03	-0.14	**0.89**	-0.04	-0.15	**0.93**	0.64	0.26	-0.35	0.00	-0.33	-0.24	-0.32	-0.22	-0.33	-0.32	**-0.94**	**0.91**	-0.19	-0.30
Hay/dried grass_A	-0.22	-0.19	-0.13	-0.29	-0.16	0.31	**0.96**	**0.95**	0.30	**0.95**	-0.15	0.15	0.65	**0.88**	**0.92**	-0.54	**0.93**	0.67	**0.92**	0.63	**0.93**	**0.93**	0.24	0.21	0.56	**0.84**
SWEET	0.04	0.06	0.62	0.70	0.54	**0.83**	0.56	0.48	**0.91**	0.55	0.16	0.76	**0.85**	0.75	0.30	-0.01	0.32	0.30	0.33	0.30	0.32	0.33	-0.53	0.78	0.29	0.33
ASTRINGENT	0.33	0.30	-0.17	-0.22	-0.08	-0.72	**-0.91**	**-0.85**	-0.73	**-0.90**	0.17	-0.59	**-0.94**	**-0.99**	-0.72	0.51	-0.74	-0.42	-0.71	-0.39	-0.74	-0.74	0.22	-0.64	-0.33	-0.60
BITTER	0.31	0.28	-0.03	-0.14	0.04	-0.55	**-0.95**	**-0.91**	-0.58	**-0.95**	0.10	-0.41	**-0.83**	**-0.98**	**-0.81**	0.48	**-0.84**	-0.53	**-0.81**	-0.49	**-0.84**	**-0.84**	0.03	-0.47	-0.42	-0.71
SOUR	0.25	0.23	-0.39	-0.59	-0.28	**-0.85**	-0.71	-0.63	**-0.90**	-0.71	0.03	-0.77	**-0.95**	**-0.89**	-0.46	0.28	-0.48	-0.25	-0.46	-0.23	-0.48	-0.49	0.48	-0.80	-0.18	-0.37
SALTY	-0.29	-0.26	-0.01	0.01	-0.07	0.51	**0.99**	**0.96**	0.54	**0.98**	-0.07	0.36	**0.81**	**0.99**	**0.87**	-0.47	**0.89**	0.58	**0.87**	0.54	**0.89**	**0.89**	0.04	0.42	0.47	0.77
THICK MOUTHFEEL	-0.29	-0.26	0.04	0.05	-0.02	0.56	**0.97**	**0.94**	0.58	**0.97**	-0.10	0.41	**0.85**	**0.99**	**0.84**	-0.48	**0.86**	0.56	**0.84**	0.52	**0.86**	**0.86**	-0.02	0.47	0.46	0.74
Fynbos-floral_F	0.31	0.29	-0.24	-0.38	-0.14	-0.75	**-0.83**	-0.76	-0.75	**-0.82**	0.25	-0.64	**-0.93**	**-0.94**	-0.61	0.53	-0.64	-0.36	-0.61	-0.32	-0.64	-0.64	0.31	-0.69	-0.26	-0.52
Rose geranium_F	0.40	0.38	-0.38	-0.66	-0.24	**-0.91**	-0.59	-0.48	**-0.93**	-0.58	0.17	**-0.86**	**-0.95**	-0.80	-0.31	0.36	-0.33	-0.01	-0.29	0.00	-0.33	-0.33	0.62	**-0.89**	0.05	-0.16
Woody_F	0.60	0.58	-0.09	-0.67	0.06	-0.72	-0.51	-0.41	-0.73	-0.50	0.22	-0.68	-0.78	-0.70	-0.27	0.42	-0.31	0.13	-0.23	0.17	-0.31	-0.30	0.48	-0.71	0.23	-0.07
Date pudding_F	-0.20	-0.21	0.68	**0.82**	0.54	**0.97**	0.15	0.03	**0.93**	0.14	-0.40	**1.00**	0.80	0.43	-0.19	-0.34	-0.15	-0.28	-0.18	-0.27	-0.15	-0.15	**-0.94**	**0.99**	-0.28	-0.25
Hay/dried grass_F	-0.06	-0.08	0.63	0.48	0.53	**0.84**	0.22	0.13	0.72	0.21	-0.65	**0.84**	0.73	0.42	-0.07	-0.57	-0.03	-0.07	-0.04	-0.07	-0.03	-0.03	-0.74	**0.84**	-0.07	-0.06

A indicates aroma attribute and F indicates flavor attribute

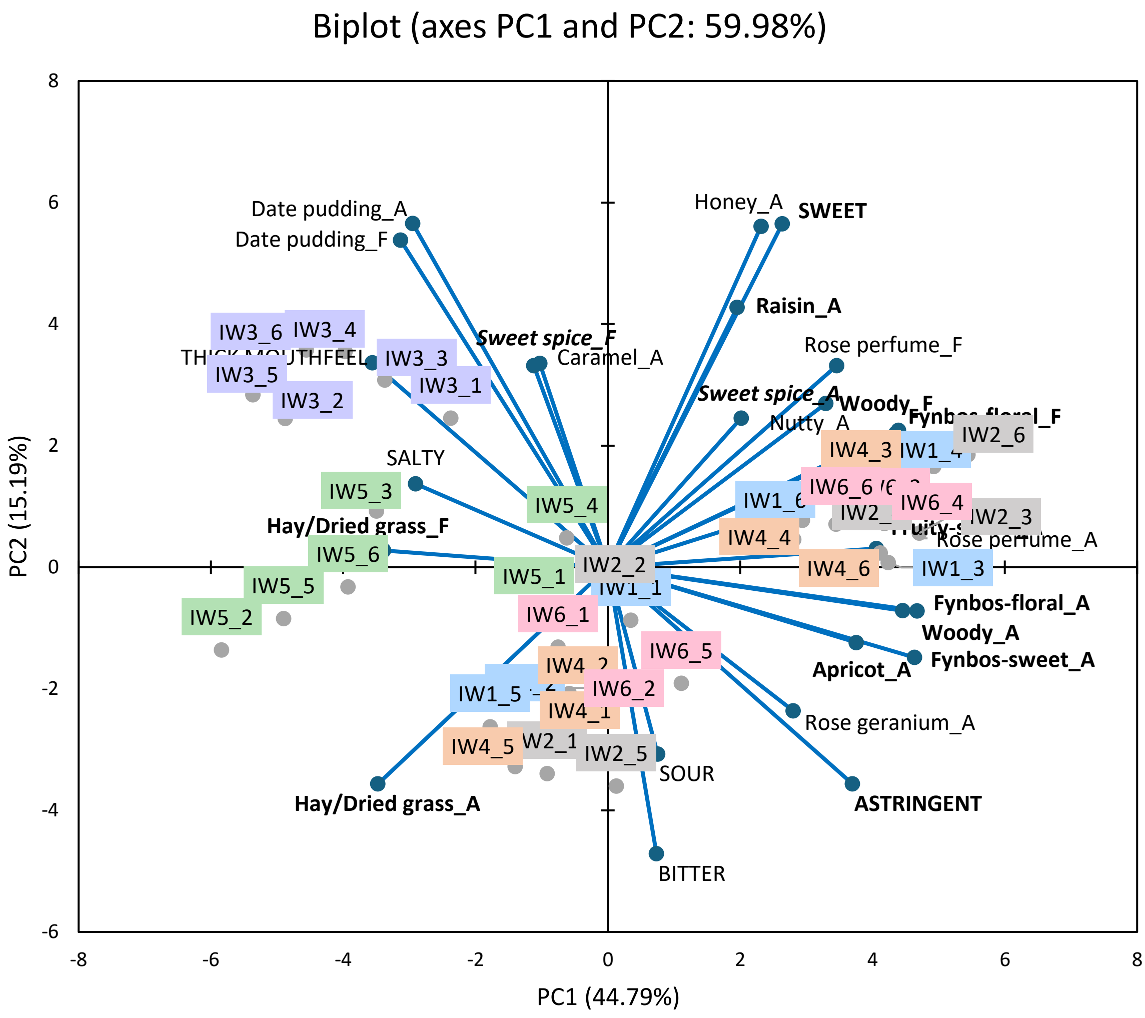

Figure S1 Principal component analysis bi-plot representing the association between all sensory attributes and ‘cup-of-tea’ *Cyclopia intermedia* infusions prepared with water from different sources (n=6). Sample labels indicate *C. intermedia* (I) infusion, followed by the water sample used to prepare the infusion (W1-W6), and lastly the *C. intermedia* sample number (IW1_1 = infusion prepared with W1 and *C. intermedia* sample 1). A indicates aroma attribute and F indicates flavor attribute. Attributes in **bold** font are generic honeybush sensory attributes, while those in ***italic bold*** font are defining characteristics of the specific species. W1, deionized water; W2, bottled still spring water from Ceres, South Africa; W3, bottled still spring water from Normandien, South Africa; W4, bottled reverse osmosis water from Oasis, Stellenbosch, South Africa; W5, brackish borehole water from a farm in Greyton, South Africa; W6, tap water from Stellenbosch, South Africa.

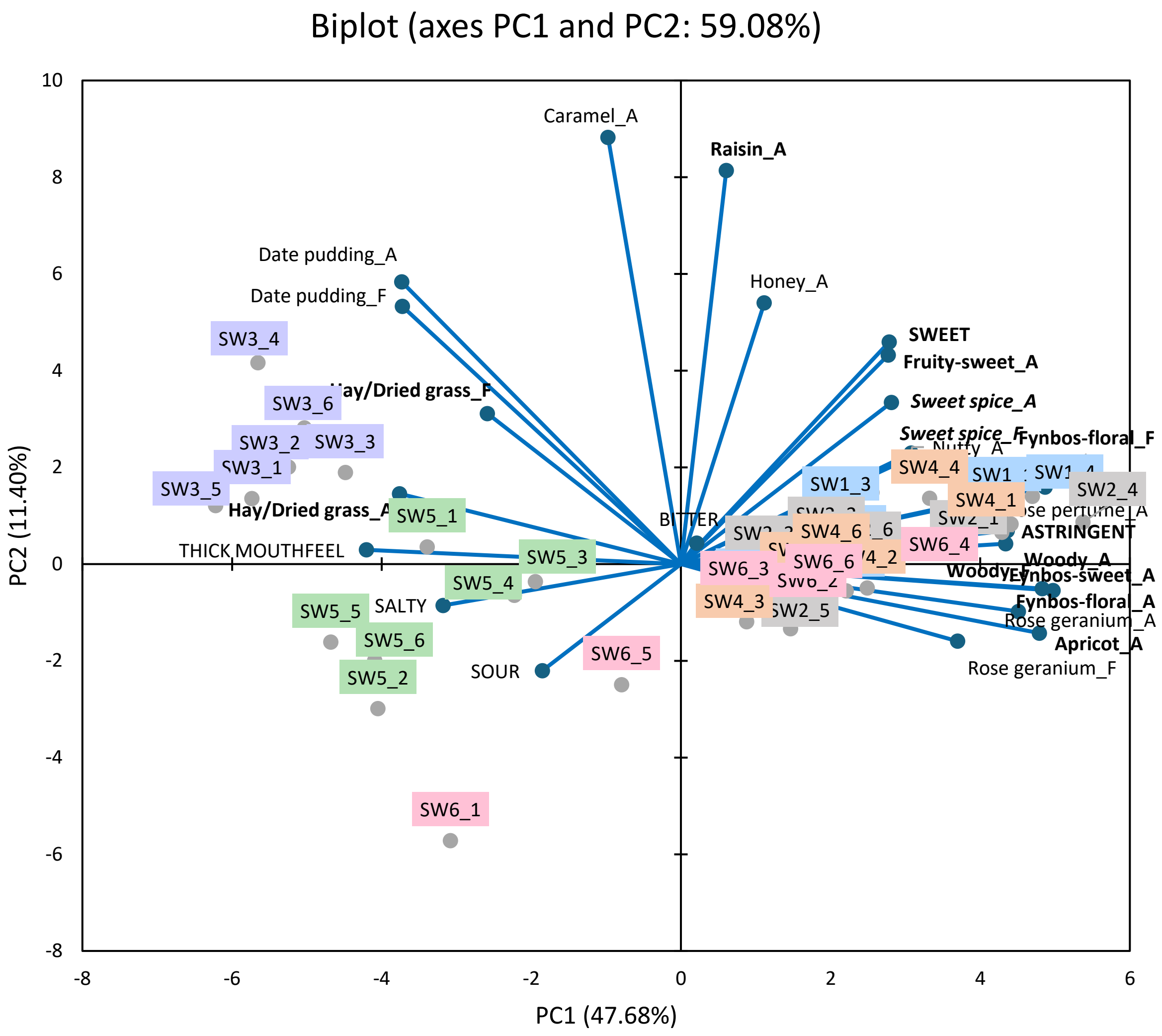

Figure S2 Principal component analysis bi-plot representing the association between all sensory attributes and 'cup-of-tea' *Cyclopia subternata* infusions prepared with water from different sources (n=6). Sample labels indicate *C. subternata* (S) infusion, followed by the water sample used to prepare the infusion (W1-W6), and lastly the *C. subternata* sample number (SW1_1 = infusion prepared with W1 and *C. subternata* sample 1). A indicates aroma attribute and F indicates flavor attribute. Attributes in **bold** font are generic honeybush sensory attributes, while those in ***italic bold*** font are defining characteristics of the specific species. W1, deionized water; W2, bottled still spring water from Ceres, South Africa; W3, bottled still spring water from Normandien, South Africa; W4, bottled reverse osmosis water from Oasis, Stellenbosch, South Africa; W5, brackish borehole water from a farm in Greyton, South Africa; W6, tap water from Stellenbosch, South Africa.

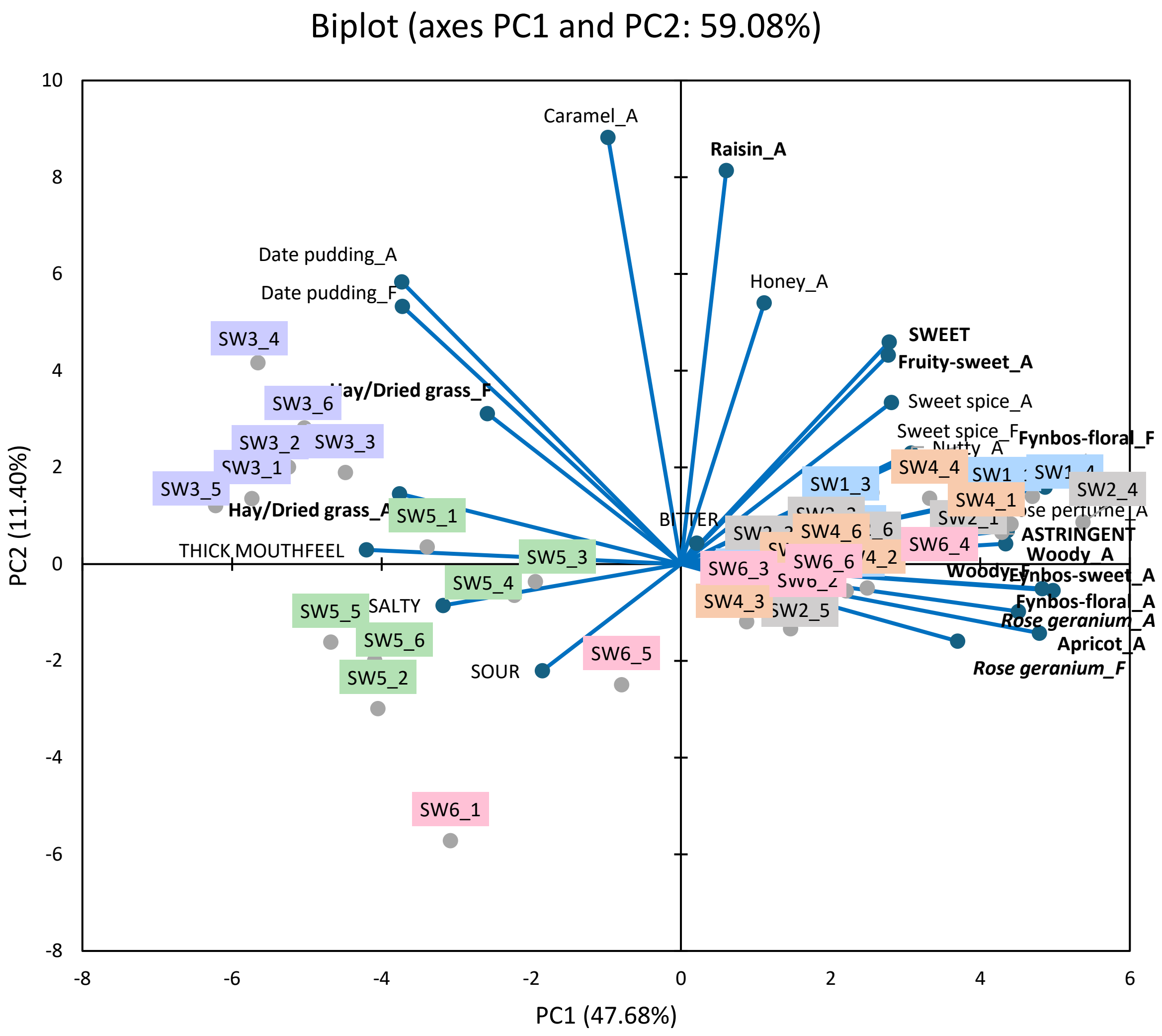

Figure S3. Principal component analysis bi-plot representing the association between all sensory attributes and 'cup-of-tea' *Cyclopia genistoides* infusions prepared with water from different sources (n=6). Sample labels indicate *C. genistoides* (G) infusion, followed by the water sample used to prepare the infusion (W1-W6), and lastly the *C. genistoides* sample number (GW1_1 = infusion prepared with W1 and *C. genistoides* sample 1). A indicates aroma attribute and F indicates flavor attribute. Attributes in **bold** font are generic honeybush sensory attributes, while those in ***italic bold*** font are defining characteristics of the specific species. W1, deionized water; W2, bottled still spring water from Ceres, South Africa; W3, bottled still spring water from Normandien, South Africa; W4, bottled reverse osmosis water from Oasis, Stellenbosch, South Africa; W5, brackish borehole water from a farm in Greyton, South Africa; W6, tap water from Stellenbosch, South Africa.

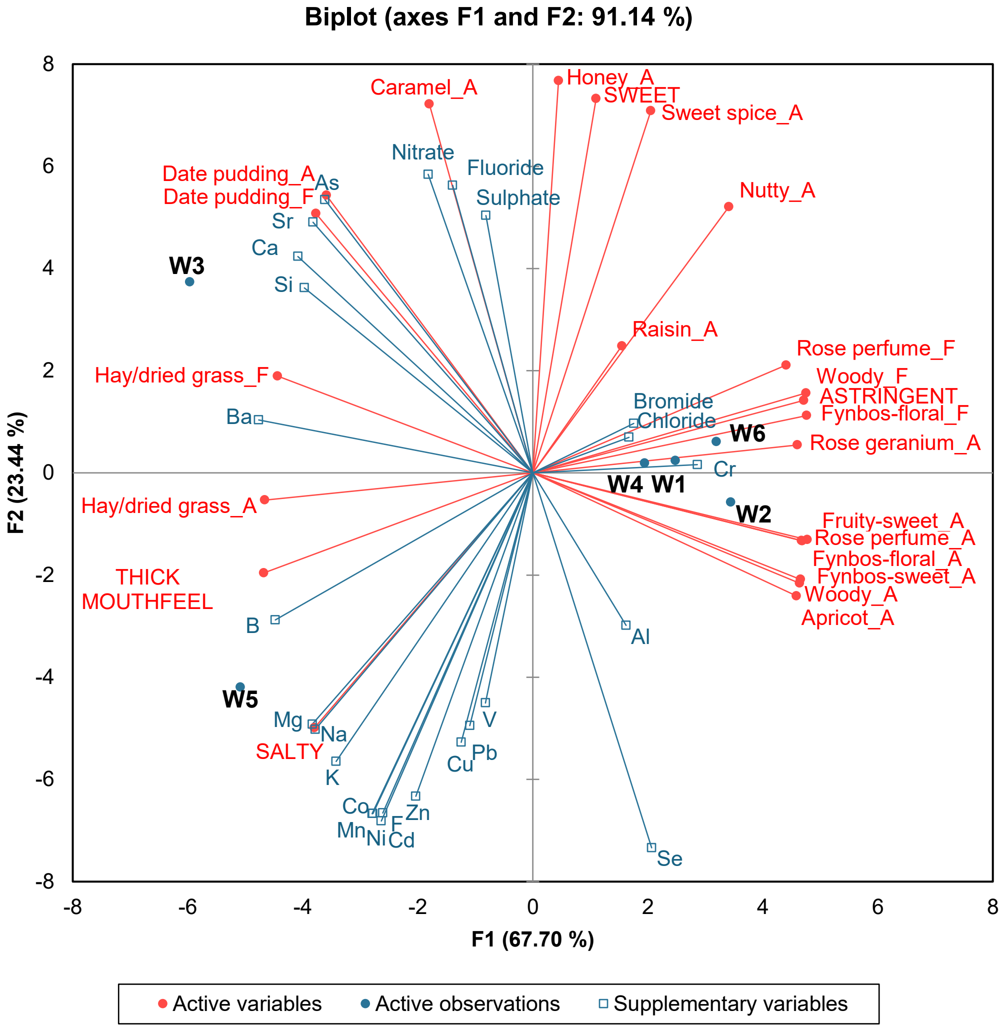

Figure S4 Principal component analysis bi-plot representing the association between the mean intensities of sensory attributes and 'cup-of-tea' *Cyclopia intermedia* infusions prepared with water from different sources (n=6) with the anion and mineral composition of the water samples included as supplementary variables. A indicates aroma attribute and F indicates flavor attribute. W1, deionized water; W2, bottled still spring water from Ceres, South Africa; W3, bottled still spring water from Normandien, South Africa; W4, bottled reverse osmosis water from Oasis, Stellenbosch, South Africa; W5, brackish borehole water from a farm in Greyton, South Africa; W6, tap water from Stellenbosch, South Africa.

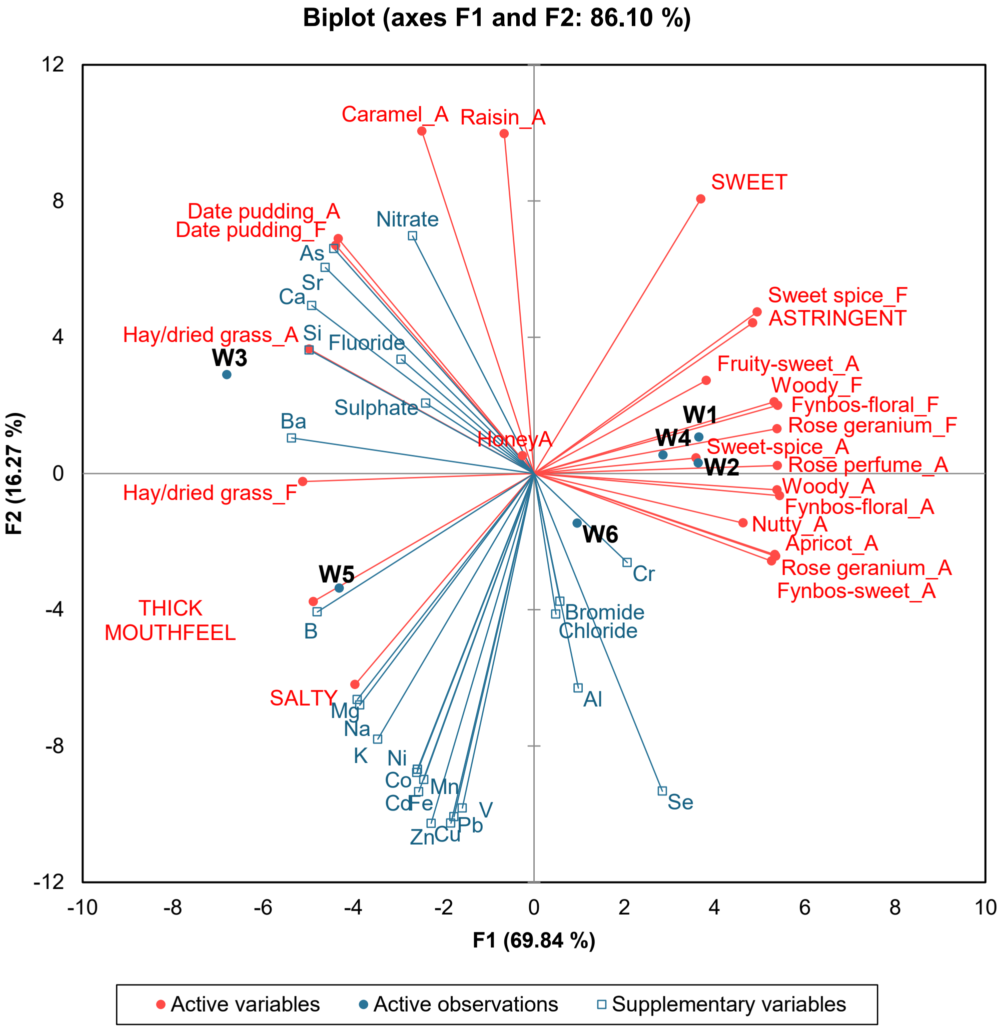

Figure S5 Principal component analysis bi-plot representing the association between the mean intensities of sensory attributes and 'cup-of-tea' *Cyclopia subternata* infusions prepared with water from different sources (n=6) with the anion and mineral composition of the water samples included as supplementary variables. A indicates aroma attribute and F indicates flavor attribute. W1, deionized water; W2, bottled still spring water from Ceres, South Africa; W3, bottled still spring water from Normandien, South Africa; W4, bottled reverse osmosis water from Oasis, Stellenbosch, South Africa; W5, brackish borehole water from a farm in Greyton, South Africa; W6, tap water from Stellenbosch, South Africa.

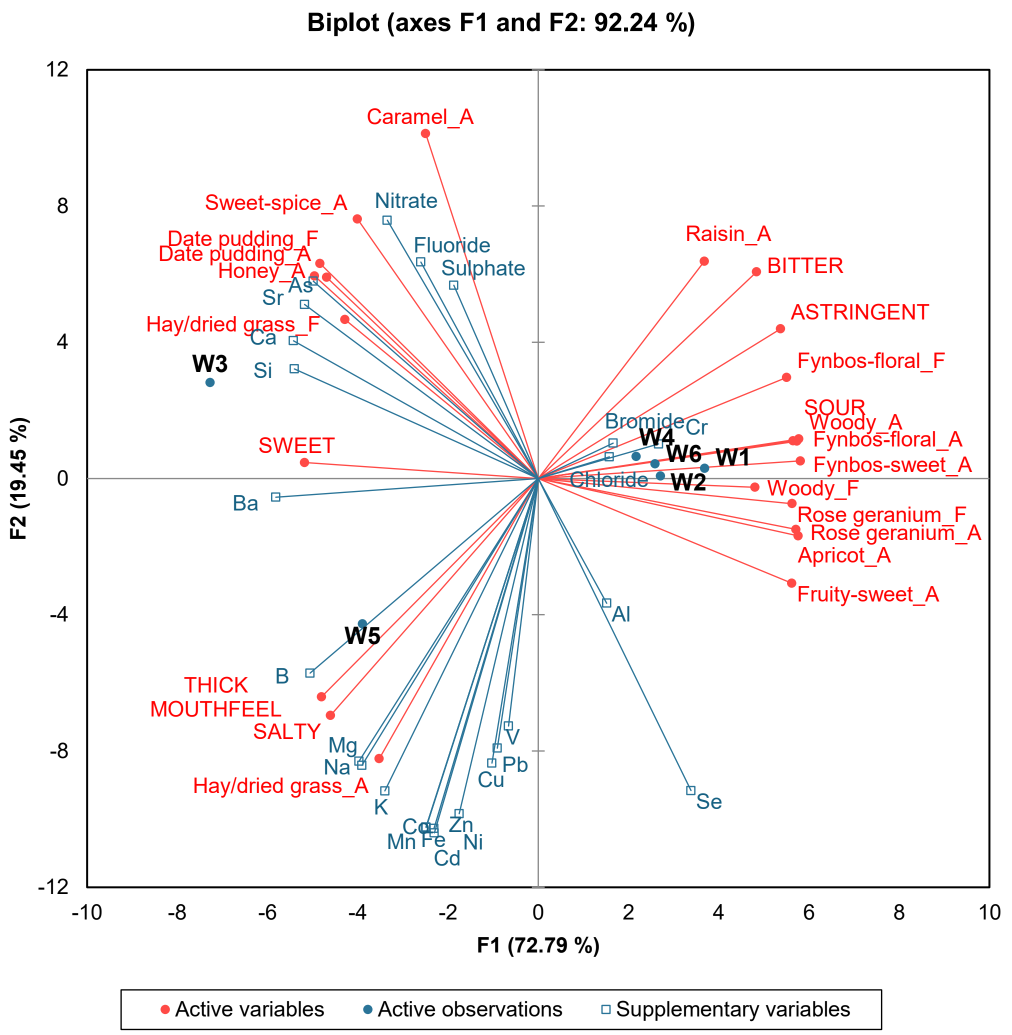

Figure S6 Principal component analysis bi-plot representing the association between the mean intensities of sensory attributes and 'cup-of-tea' *Cyclopia genistoides* infusions prepared with water from different sources (n=6) with the anion and mineral composition of the water samples included as supplementary variables. A indicates aroma attribute and F indicates flavor attribute. W1, deionized water; W2, bottled still spring water from Ceres, South Africa; W3, bottled still spring water from Normandien, South Africa; W4, bottled reverse osmosis water from Oasis, Stellenbosch, South Africa; W5, brackish borehole water from a farm in Greyton, South Africa; W6, tap water from Stellenbosch, South Africa.
